# Dissolution of Fe(III)-rich basaltic glass in microbial cultures of iron-reducing microorganisms

**DOI:** 10.3389/fmicb.2026.1814551

**Published:** 2026-06-01

**Authors:** Elena Yunda, Aurore Gorlas, François Guyot, Denis Testemale, Damien Daval

**Affiliations:** 1 ISTerre, CNRS, IRD, Université Gustave Eiffel, Université Grenoble Alpes, Université Savoie Mont Blanc, Grenoble, France; 2 CEA, CNRS, Institute for Integrative Biology of the Cell (I2BC), Université Paris-Saclay, Gif-sur-Yvette, France; 3 IMPMC Muséum National d'Histoire Naturelle, 4 Place Jussieu, Paris, France; 4 CNRS, Grenoble INP, Institut Néel, Université Grenoble Alpes, Grenoble, France; 5 FAME and FAME-UHD Beamlines, European Synchrotron Radiation Facility, Grenoble, France

**Keywords:** basaltic glass, dissolution rate, glass alteration, iron-reducing microorganisms, *Pyrobaculum islandicum*, *Thermus scotoductus*, vertical scanning interferometry

## Abstract

The aim of this study was to investigate the contribution of Fe(III)-reducing microorganisms to the dissolution rates of Fe(III)-rich synthetic basaltic glass. Hyperthermophilic archaeon *Pyrobaculum islandicum* and thermophilic bacterium *Thermus scotoductus* were incubated for 7 or 15 days with basaltic glass, and the surface retreat of the glass was determined using vertical scanning interferometry. *Pyrobaculum islandicum* was shown to enhance basaltic glass dissolution rate two-fold compared to abiotic controls in 7-days incubations. However, this effect was only 1.3-fold in 15-days incubations. In contrast, *Thermus scotoductus* did not impact or slightly inhibited the dissolution. Accordingly, alteration microstructures of the surface were visible only with *Pyrobaculum islandicum* cultures which promoted the formation of (Al, P, S)-rich layers thickening with time at the surface of the glass as well as of (Fe, S)-bearing crystals. To identify the underlying mechanisms that could explain such differences, Fe(III) reduction assays were performed in cultures with Fe(III) citrate, revealing higher rates of Fe(III) reduction in incubations with *Pyrobaculum islandicum* compared to those with *Thermus scotoductus*. Collectively, these findings suggest that Fe(III)-reducing microorganisms can enhance basaltic glass dissolution, but that the process could be limited by the rate of Fe(III) reduction and by surface alteration products.

## Introduction

Silicate weathering is a fundamental process contributing to shaping the lithosphere and controlling the long-term carbon cycle (Berner et al., 1983). The dissolution of Ca- and Mg-rich silicate phases consumes carbon dioxide (CO_2_) via the formation of dissolved bicarbonate ions and secondary carbonate minerals. This process forms a basis for various strategies of enhanced removal of atmospheric carbon dioxide that were in focus of recent research to mitigate climate change (Campbell et al., 2022; Beerling et al., 2020; Timmermann et al., 2025).

Basalts have a major contribution to silicate weathering and CO_2_ consumption (Dessert et al., 2003; Navarre-Sitchler and Brantley, 2007; Beerling et al., 2020). Their reactivity depends on multiple physicochemical parameters, including temperature, pH, surface area, solution composition and saturation index (Brantley et al., 2023; Gudbrandsson et al., 2011; Gislason and Oelkers, 2003; Navarre-Sitchler and Brantley, 2007; Berger et al., 1994; Bas-Lorillot et al., 2026). While these abiotic factors have been extensively studied, the biotic contribution to silicate weathering remains less well constrained (Brantley et al., 2023; Timmermann et al., 2025). Numerous studies revealed diverse microbial communities inhabiting basaltic subsurface environments (Bergsten et al., 2021; Bas-Lorillot et al., 2025; Ivarsson et al., 2015, Ivarsson and Neubeck, 2016; Fisk et al., 2003; Lysnes et al., 2004; Mason et al., 2009; Jørgensen and Zhao, 2016; Zhang et al., 2016; Santelli et al., 2008). Globally, more than half of bacterial and archaeal cells on Earth are estimated to live in the subsurface, where porous basalt represents a significant microbial habitat (Flemming and Wuertz, 2019; Ivarsson et al., 2015). Yet, microbial cell densities in basaltic rocks are typically low [e.g., ~10^4^ cells per cm^3^ or g of oceanic subsurface basalt (Jørgensen and Zhao, 2016; Zhang et al., 2016)], making studies of these communities and their potential impact on basalt alteration particularly challenging.

Documented mechanisms of microbial influence on silicate weathering include changes in pH and production of organic acids and chelating molecules (Barker et al., 1997; Barker et al., 1998; Buss et al., 2007; Weaver et al., 2021; Valbi et al., 2023). For example, siderophore production by bacteria may enhance the dissolution of Fe(III)-bearing basaltic glass (Perez, Rossan et al., 2019). The attachment of cells to the silicate surface is therefore not a prerequisite for its alteration, although it has been shown to enhance the dissolution in some cases (Ahmed and Holmström, 2015). In subsurface environments, most microbial cells are thought to be attached to the surface of rocks (Flemming and Wuertz, 2019). In parallel, many studies have revealed pits and microchannels formed in silicates, hypothetically attributed to localized microbial activity (Buss et al., 2007; Chen et al., 2014; Nikitczuk et al., 2016; Wacey et al., 2017). These features were the subject of high-resolution microscopy and chemical studies that showed the differences in the composition of altered areas vs. surrounding rock (Benzerara et al., 2007; Knowles et al., 2012; Wacey et al., 2017). A model of proton flux through a microbial cell to the surface of basaltic glass, and release of cations from the glass to balance the charges has been proposed (Fisk et al., 2019).

Despite many advances in understanding microbe-silicate interactions, the impact of cells remains not conclusive as (i) pits and microchannels can be formed abiotically or without evidence of organic carbon (Fisk et al., 2013; Wacey et al., 2017), and (ii) the impact of bacteria on silicate dissolution can be negligible (Shirokova et al., 2012; Stockmann et al., 2012). For example, species of the same genus of bacteria showed contrasting effects on the dissolution of basaltic glass, where one strain of *Pseudomonas* (*P. fluorescens*) increased the dissolution rate (Chen et al., 2014), while another strain (*P*. *reactans*) did not have impact or even slightly decreased the dissolution rate (Stockmann et al., 2012). Hence, microbial influence on basalt weathering can vary between strains, and the factors underlying this difference are not fully clear.

Studies on oceanic subsurface basalt revealed microorganisms represented predominantly by bacteria [e.g., *Pseudomonadota* (Jørgensen and Zhao, 2016; Zhang et al., 2016; Bergsten et al., 2021)], and a minor fraction of archaea [e.g., Crenarchaeota (Jørgensen and Zhao, 2016; Bergsten et al., 2021)]. Many of the identified microorganisms belong to thermophiles (Bergsten et al., 2021) and are involved in both heterotrophic and chemolithotrophic metabolisms (Stevens, 1997; Lau et al., 2016; Zhang et al., 2016; Bergsten et al., 2021). Considering the latter metabolic pathway, estimations from thermodynamic calculations of bioavailable energy proposed Fe(II) oxidation as one of the main routes to source energy for microbial growth in basaltic settings (Bach and Edwards, 2003; Bach, 2016). These findings were supported by experimental observations of Fe-oxidizing bacteria in basalt layer of oceanic crust (Zhang et al., 2016) and on synthetic basaltic glass incubated on surface sediments of the Mid Atlantic Ridge abyssal plain (Henri et al., 2016). Incubation of basaltic glass with cultures of single bacterial isolates also confirm that Fe(II) serves nutritive functions for cells (Edwards et al., 2003; Stranghoener et al., 2018).

In parallel, it was also shown that as basalt undergoes alteration and Fe(II) becomes less available, more diverse metabolisms are potentially employed for harvesting energy, resulting in a higher microbial diversity (Santelli et al., 2008, 2009). Thus, bioenergetic and thermodynamic calculations predict H_2_ as a prevalent electron donor in altered basalts, as opposed to Fe(II) in younger rocks (Türke et al., 2015). Recent studies of a basaltic glacial catchment in Iceland suggest H_2_/Fe(III) redox couple to be a driving force for microbial colonization in these environments (Dunham et al., 2021, 2023). In accordance with these findings, enrichment cultures of basaltic sediments from glacial catchments and sea-floor basalt show presence of Fe(III)-reducing bacteria (Dunham et al., 2021; Lysnes et al., 2004). Hence, microbial processes could significantly contribute to iron redox cycling in basaltic environments. While previous studies suggest microbial Fe(II) oxidation contribute to alteration reactions (Henri et al., 2016; Santelli et al., 2008), the role of Fe(III)-reducing cell activity in basalt weathering remains ambiguous.

Iron redox state also exerts a strong control on the reactivity of Fe-bearing silicate minerals (Sissmann et al., 2013; Saldi et al., 2013, 2015) and glasses (Paul and Zaman, 1978). The formation of passivating amorphous [Fe(III)-Si]-rich layers at the surface of dissolving silicates has been proposed as one of the possible mechanisms responsible for the well-documented decrease in silicate weathering rates with time. As a consequence, any agent likely to promote Fe(III) reduction could therefore contribute to surface depassivation and enhanced substrate dissolution. For instance, in a study of olivine dissolution and carbonation reactions under oxic conditions, the formation of tightly bound Fe(III)-oxides within an amorphous silica-rich surface layer was found to inhibit olivine dissolution. Conversely, under micro-oxic conditions, Fe(II) mobilization was observed, concomitantly with the resumption of olivine dissolution (Saldi et al., 2015). Similarly, we recently showed that the dissolution rate of basaltic glass is higher under micro-oxic conditions (Bas-Lorillot et al., 2026). Therefore, investigating the kinetics and mechanisms of basaltic glass weathering in reducing systems with microorganisms capable of electron transfer to Fe(III) phases is of prime interest for understanding silicate weathering in natural settings.

Experimental investigation of such processes requires model systems with well-controlled compositions and redox states. Synthetic basaltic glass provides a convenient approach, as it can be designed with homogeneous Fe(III) distribution and content, including biologically nutritive elements (Henri et al., 2016; Stranghoener et al., 2018; Perez Rossano et al., 2019).

In addition, microbially-mediated dissolution studies require complex and concentrated nutritive media, in which the monitoring of dissolution through cation release can be challenging. Vertical scanning interferometry (VSI) provides a methodological approach to visualize large (~cm^2^) surface areas with a nanometer-scale vertical resolution. By masking a part of the basaltic glass surface, it is possible to compare the exposed surface of the basaltic glass with its unreacted counterpart. This method was successfully applied to quantify microbially-mediated dissolution rates of iron-silicate glass (Buss et al., 2007), silicate minerals (Wild et al., 2021; Quirk et al., 2012), and carbonate minerals (Davis and Lüttge, 2005; Stigliano Benzerara et al., 2025; Fischer et al., 2012).

In this work, the ability of two iron-reducing microorganisms to enhance basaltic glass reactivity was estimated. We determined dissolution rates and surface alteration of synthetic basaltic glass after incubation with microorganisms and compared the rates to abiotic controls of the respective nutritive media. The microorganisms used were the hyperthermophilic archaeon *Pyrobaculum islandicum* and the thermophilic bacterium *Thermus scotoductus*. These strains belong to genera previously identified in borehole samples of underground water in Iceland (Bas-Lorillot et al., 2025). The conditions for growth of these strains reflect diverse scenarios that could occur in the subsurface, including relatively low temperature and hypoxic conditions (*T. scotoductus*) and contrasting high temperature and strong reducing environments (*P. islandicum*). The basaltic glass used for the incubations with microorganisms was synthesized under conditions favoring Fe(III), mimicking the oxidation experienced by aged basaltic glass. Basaltic glass surface was exposed to nutritive media with or without microbial cultures and dissolution rates were quantified using VSI analysis. Complementary scanning electron microscopy and energy-dispersive X-ray spectroscopy (SEM–EDX) analyses were used to characterize chemical alterations and secondary phases at the glass surface. In parallel experiments, iron reduction rates were quantified *in situ* using X-ray absorption spectroscopy, where Fe(III) citrate was added as a sole electron acceptor. Fe(III) citrate was used as a substitute to basaltic glass in these experiments to provide a soluble substrate, commonly used to study dissimilatory Fe(III) reduction by microorganisms, including *P. islandicum* and *T. scotoductus* (Vargas et al., 1998; Kieft et al., 1999).

The hypothesis that we wanted to test in this work was that microbial Fe(III) reduction could depassivate the basaltic glass surface and thereby enhance its dissolution. The findings of this work allow for a better understanding of microbial contribution to basaltic glass alteration and suggest that high rates of microbial Fe(III) reduction are associated with enhancement of basaltic glass dissolution.

## Materials and methods

### Microbial strains and culture media

*Pyrobaculum islandicum* DSM 4184 was purchased from DSMZ, Braunschweig, Germany and grown in M390 medium (DSMZ) lacking FeCl_3_ and containing per liter: 1.3 g (NH_4_)_2_SO_4_, 0.28 g KH_2_PO_4_, 0.25 g MgSO_4_ · 7 H_2_O, 0.07 g CaCl_2_ · 2 H_2_O, 0.5 g trypticase peptone (Carl ROTH), 0.2 g yeast extract (Sigma-Aldrich), and 10 mL of Allen’s trace element solution (per liter: 180 mg MnCl_2_ · 4 H_2_O, 450 mg Na_2_B_4_O_7_ · 10 H_2_O, 22 mg ZnSO_4_ · 7 H_2_O, 5 mg CuCl_2_ · 2 H_2_O, 3 mg Na_2_MoO_4_ · 2 H_2_O, 3 mg VOSO_4_ · 2 H_2_O, 1 mg CoSO_4_ · 7 H_2_O). Trypticase peptone and yeast extract served as electron donors in the media. Anaerobic conditions were maintained using ~7 mL/L of 10% (w/v) Na_2_S · 9 H_2_O aqueous stock solution, and monitored using sodium resazurin (0.5 mL/L of 0.1% w/v stock solution). Sodium thiosulfate (2 g/L Na_2_S_2_O_3_ · 5 H_2_O) was used as an electron acceptor, except when basaltic glass was added in the bottle with the medium. For routine culture growth, 15 mL of the autoclaved medium was aseptically transferred into 50-mL crimp-sealed vials, in which 150 μL of 20% (w/v) filter-sterilized stock solution of sodium thiosulphate was added. The headspace was flushed with N_2_, Na_2_S solution was added and media were kept for ~15 min to establish reducing conditions before adding the cell culture into the medium (1:50 v/v). Cultures were grown for 48–50 h at 93–95 °C until the cell density of ~10^7^ cells·mL^−1^ (determined with flow cytometry, as described below), and used the same day for the incubation with the basaltic glass.

*Thermus scotoductus* DSM 8553 was purchased from DSMZ, Braunschweig, Germany. The medium for *T. scotoductus* cultivation was based on ATCC 461 (Balkwill et al., 2004) and DSMZ 878 media, and contained per liter: 0.04 g CaSO_4_ · 2 H_2_O, 0.1 g MgSO_4_ · 7 H_2_O, 0.5 g KH_2_PO_4_, 4.3 g Na_2_HPO_4_ · 12 H_2_O, 0.1 g nitrilotriacetic acid, 1 g trypticase peptone, 1 g yeast extract, and 0.5 mL of the trace element solution (per liter: 0.5 mL H_2_SO_4_, 2.28 g MnSO_4_ · H_2_O, 0.5 g ZnSO_4_ · 7 H_2_O, 0.5 g H_3_BO_3_, 0.025 g CuSO_4_ · 5 H_2_O, 0.025 g Na_2_MoO_4_ · 2 H_2_O, 0.053 g CoSO_4_). Trypticase peptone and yeast extract served as electron donors in the media. Cell cultures were routinely maintained by culture transfer (1:50 v/v) into 20 mL of the aerobic nutritive medium in 50-mL Erlenmeyer flasks and incubation at 60 °C for 46–48 h until the cell density of ~10^8^ cells·mL^−1^. Ultrapure water (18.2 MΩ·cm) was used for all media preparation.

### Microbial suspensions and media for incubation with basaltic glass

For both microorganisms, microbial suspensions for incubation with basaltic glass were prepared by harvesting cells at the late exponential growth phase (~48 h) using centrifugation at 4000 rpm for 15 min, and resuspending in the fresh incubation medium.

Media for *P. islandicum*. The incubation medium composition was identical to culture medium composition, except that for promoting microbial Fe(III) reduction in basaltic glass, electron acceptors were omitted from the incubation media. Thus, thiosulphate was not present in the incubation medium of *P. islandicum*, and *P. islandicum* cells were washed once with the incubation medium before exposure to basaltic glass to minimize traces of thiosulphate left after culture growth.

Media for *T. scotoductus*. The incubation medium composition was identical to culture medium composition, except that the incubation medium was purged with N_2_ to remove dissolved O_2_ (≤0.2 ppm, Winkler kit, Hanna instruments). *T. scotoductus* cells harvested by centrifugation were resuspended in the incubation medium without preliminary washing step, as there were no electron acceptors present in the medium, apart from O_2_ that was removed during flushing procedure before the start of the incubation.

The volume of the media for incubation was (60 ± 1) mL for *P. islandicum* and (58 ± 2) mL for *T. scotoductus*. A large medium volume relatively to sample dimensions was selected to minimize the contribution of Si release from borosilicate to the basaltic glass dissolution rate. Si concentrations and other major elements concentrations were monitored using ICP-OES Agilent 5,800 VDV in filtered (0.2 μm) samples acidified with HNO_3_ (Supplementary Table S1; Supplementary Figure S1-S2). The pH of the media (T ≈ 22 °C) at the start of the incubation was 6.7 ± 0.1 for *P. islandicum,* and 7.5 ± 0.1 for *T. scotoductus*. The temperature of the incubation was 93 °C and 60 °C, respectively. Further details on the incubation procedure for *P. islandicum* and *T. scotoductus* are presented below.

### Microbial cell counting

To monitor cell number in *P. islandicum* cultures, cells were stained using 0.15% (v/v) of 3.34 mM SYTO 9 stock solution in DMSO (Invitrogen™) for 15 min in the dark and counted using epifluorescence microscope (ZEISS Axio Vert. A1). A series of images was recorded on culture droplets deposited either on bare (2 μL culture drop dried at 90 °C) or agar-coated glass slides (31 μL culture drop covered with 20×20 mm coverslip and sealed with the mixture of paraffin and paraffin oil (Pfennig and Wagener, 1986)). The images were processed in BiofilmQ software (Hartmann et al., 2021) for quantification of cell number based on image segmentation into squares with the side length comparable to the length of *P. islandicum* (2.5–2.8 μm). Alternatively, cells were counted using Trainable Weka segmentation plugin in Fiji (Schindelin et al., 2012). The microscopy results were compared with counts obtained from flow cytometry measurements. For flow cytometry analyses, cells were fixed with glutaraldehyde (final concentration 2.5%), immediately flash-frozen in liquid nitrogen, and stored at −80 °C. Samples were stained with SYBR Green I (Invitrogen™, 10,000x stock solution). After incubation for 15 min at room temperature in the dark, samples were analyzed using CytoFLEX flow cytometer (Beckman Coulter). Data acquisition and processing were performed using the CytExpert software. For *T. scotoductus*, the cell growth was routinely followed using optical density measurements at 600 nm (Agilent Cary 3,500 UV–Vis). To convert optical density values to cell numbers, a series of *T. scotoductus* cultures with known optical density values was measured using flow cytometry using the same parameters as for *P. islandicum* cells.

### Cell number in microbial suspensions with basaltic glass

Microbial suspensions for the incubation with basaltic glass were prepared from freshly grown 48-h old cultures that contained (0.9 ± 0.4) · 10^7^ cells·mL^−1^ and (2 ± 0.7) · 10^8^ cells·mL^−1^ for *P. islandicum* and *T. scotoductus*, respectively (Supplementary Figure S3). To obtain a similar concentration of cells in the incubations with both microorganisms, the cultures containing *T. scotoductus* were diluted 25 times in the first experiment that lasted for 7 days. As *T. scotoductus* did not induce changes in the dissolution rate of the basaltic glass (described below), we tested the conditions with higher *T. scotoductus* concentration. We increased the number of *T. scotoductus* cells in the incubation for all subsequent experiments, by diluting freshly-grown cultures only 5 times. Thus, the cell concentration in incubations was approximately 1 · 10^7^ cell·mL^−1^ and 4 · 10^7^ cells·mL^−1^ for *P. islandicum* and *T. scotoductus*, respectively. The growth of cells was monitored before the start of each experiment using epifluorescence microscopy and optical density measurements for *P. islandicum* and *T. scotoductus*, respectively, and the number of cells was found to be consistent between all incubations within a 1.5 factor of difference. Over time of the incubation, the biomass growth is not expected because the incubation medium lacked thiosulfate, while Fe(III) in basaltic glass would not sustain cell growth in a full culture volume due to a low ratio of the coupon surface area to the culture volume.

### Incubation of basaltic glass in microbial suspensions

Synthetic basaltic glass was prepared following the protocol described in (Bas-Lorillot et al., 2026). In brief, a crystalline basalt sample collected from the banks of the Hvita river, Iceland, was melted in a furnace at 1500 °C and quenched under ambient atmosphere, before being annealed at 600 °C. The Fe oxidation state of the samples made using this procedure was 25% Fe(II) – 75% Fe(III) [see Bas-Lorillot et al. (2026)]. The obtained ingots were then sliced into ~1 mm-thick disks using diamond wheel saw (Model 650, South Bay Technology) and polished (PRIMEVerre, France) to obtain coupons with a roughness S_a_ = (9 ± 4) nm, determined on 1 mm^2^ of the sample surface using 10x objective of vertical scanning interferometer (VSI, ZYGO NewView 7,300). The disks were cut using the diamond wheel saw to obtain samples with a geometric surface area of (8 ± 1) mm^2^. Before the incubation, approximately 2 mm^2^ of the coupon surface was covered with room-temperature vulcanizing glue spot that was left to cure for 24 h. The masked area was used as a reference for determining the surface retreat after the incubation experiments, as further described below.

Incubation in *P. islandicum* suspensions. Biotic experimental conditions included the setup (i) with *P. islandicum* cells and Na_2_S, and (ii) with *P. islandicum* cells and without Na_2_S. However, we noticed that the latter conditions resulted in 80% decrease in cell count overnight, as determined using flow cytometry and epifluorescence microscopy measurements. Hence, the addition of a reducing agent was necessary to maintain cells viable during the incubation and therefore, all biotic incubations presented in the study contained Na_2_S. Abiotic experimental conditions included the setup (i) without *P. islandicum* and with Na_2_S, and (ii) without *P. islandicum* and without Na_2_S. Additionally, control conditions with killed cells were included, where Na_2_S was present at the same concentration as in the biotic samples. Killed cells were prepared by harvesting, washing and resuspending cultures in the same way as for the experiment with active cultures, where the only difference was the autoclaving step of the bottles for 45 min preliminarily to the addition of basaltic glass disks. All used incubation conditions with corresponding numbers of replicates are summarized in Supplementary Table S1. Statistically significant differences between biotic and abiotic samples were analyzed using Student’s *t*-test in Microsoft Excel.

Incubation in *T. scotoductus* suspensions. Biotic experimental conditions included *T. scotoductus* suspensions, prepared in media as described above, as well as suspensions with starved cells or suspensions incubated under oxic conditions. Starved cells were prepared by the incubation of *T. scotoductus* cultures in 10 mM PIPES buffer (Kieft et al., 1999) for 48 h at 60 °C to deplete energy-reserve intracellular compounds, preliminarily to mixing with basaltic glass. The incubation at oxic conditions with *T*. *scotoductus* was performed by adding a coupon of basaltic glass at the start of the routine culture growth, described above, except that the culture was kept at 60 °C for 8 instead of 2 days to enhance the accumulation of microbially-produced pigments in the medium. As for *P. islandicum*, all used incubation conditions are summarized in Supplementary Table S1.

### Quantification of the dissolution rate of basaltic glass

**Figure 1:**
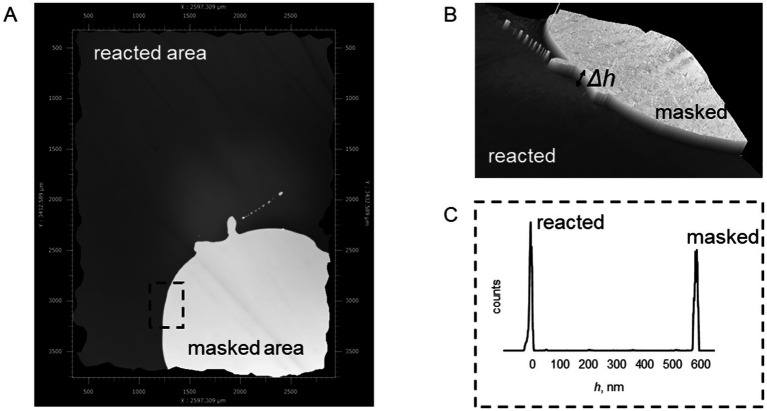
Surface retreat measurements with VSI. **(A)** Basaltic glass showing reacted and masked areas after the incubation in the microbial fluid and removal of the glue spots. **(B)** Three-dimensional view showing surface retreat Δ*h, q*uantified as **(C)** the difference in relative surface heights of the reacted and masked area outlined in **(A)**.

Dissolution rates of basaltic glass coupons were determined based on surface retreat data obtained using VSI (Perez, Daval et al., 2019). Glue spots were wiped off from sample surfaces to reveal the portion of the surface that was not exposed to the microbial fluid and as such, serving as an internal reference representing the initial topography of the sample. The unmasked portion of the samples exposed to the microbial fluid was dissolved, resulting in the surface retreat relative to the masked area (Figure 1). For the majority of the conditions used, the surface retreat, *Δh*, was determined by quantifying the relative height difference between the exposed and masked areas in (0.2 ± 0.1) mm^2^ of the sample surface area. For few samples on which it was not possible to remove secondary precipitates from vicinity of the masked area, the retreat was calculated as the lowest observed topography along the mask edge, where breaches in the secondary layers were clearly identified. The dissolution rate *r* was further determined using the following equation (Equation 1):


$$r=\frac{\mathrm{\Delta h}}{\mathrm{\Delta t}\cdot V_{m}}(\mathrm{mol}/m^{2}/s)$$ (1)

Where Δ*h* is the average surface retreat (m), Δ*t* is the incubation time (s), and *V_m_* is the molar volume of basaltic glass (4.6·10^−5^ m^3^/mol).

### Surface characterization of basaltic glass

At the end of the incubation experiments, basaltic glass samples were carefully removed from the media and dipped twice in sterile ultrapure water to remove non-adherent cells. Samples were dried and stored in air before observation with scanning electron microscopy (SEM) and Energy-dispersive X-ray spectroscopy (EDXS). To enhance the possibility of observing secondary precipitates, two batches of 100–250 μm basaltic glass powders (10 mg) were incubated in *P. islandicum* cultures (2 mL) in media of the same composition as used for the bulk basaltic glass coupons. After removal from microbial suspensions, powders were directly deposited on carbon tape and dried in air. Few basaltic glass coupons were observed in cross-section after preparation in epoxy resin. Coupons in resin were left to solidify for 24 h at room temperature under compressed air pressure to remove air bubbles, followed by polishing (final diamond particle size 1 μm). Samples were carbon-coated and imaged using Gemini Zeiss™ Ultra55 field emission gun scanning electron microscope equipped with a Bruker silicon drift detector for EDXS. A working distance of 7.5 mm and acceleration voltage of 10 kV was used as standard measurements conditions for SEM and EDXS. For observation of the extracellular matrix surrounding microorganisms, few samples were observed at a working distance of 3.8 mm and acceleration voltage of 2 kV.

Two representative samples were selected for focused ion beam (FIB) milling using a dual-beam FIB scanning electron microscope (FEI Helios 600 Nanolab system; CP2M, Marseille, France). These samples were selected to represent abiotic and biotic incubations of basaltic glass in *P. islandicum* medium containing Na_2_S. Both samples were stored in Ar glove box before coating with carbon layer. Prior to FIB milling, a protective platinum layer was deposited over the target area. Ga^+^ ion milling produced cross-section approximately 5 × 10 μm in size, lifted out with an Omniprobe 100.7 micromanipulator, and mounted on a coper mesh grid with carbon thin film for final thinning to approximately 100 nm thickness.

The lamellae were analyzed by transmission electron microscopy (TEM) using a JEOL 2100F (IMPMC, Paris, France) equipped with a field emission gun (FEG) operated at 200 kV both with parallel electron beam and in scanning probe mode (STEM) using high angle annular dark field imaging. Chemical mapping was made in STEM mode using an energy dispersive X-ray system from JEOL.

### Quantification of Fe(III) reduction by microbial strains

We attempted to quantify Fe concentration and speciation during the incubation with basaltic glass (20 mg of 100–250 μm powder in ~100 μL of microbial culture) using X-ray absorption near-edge structure (XANES) spectroscopy *in situ* and in real time, but Fe concentration remained below the detection limit within the timeframe of synchrotron measurements. Therefore, we performed experiments where basaltic glass was replaced with Fe(III) citrate, as a proof of concept for the experiments employing a synchrotron radiation to study live anaerobic cultures of hyperthermophilic microorganisms (Picard et al., 2012). Fe(II) proportion from total Fe was determined in situ using XANES with spectra recorded at short time intervals (~20 min). We then duplicated the experiment ex situ using ferrozine assay (described below), at similar conditions as those of the synchrotron experiment to verify reproducibility of the data at several time points, as well as changes in cell concentrations during the incubation with Fe(III) citrate.

*P. islandicum* and *T. scotoductus* cultures were freshly prepared for each experiment, harvested by centrifugation as described above, and resuspended in the nutritive media containing Fe(III) citrate. Fe(III) citrate (20 mM) was added as a sole electron acceptor in the nutritive media corresponding to each strain. The media were flushed with N_2_ before inoculation of both strains, and L-cysteine (0.25 mM) was added to *P. islandicum* medium as a substitute for sodium sulfide (Kashefi and Lovley, 2000; Feinberg et al., 2008). Abiotic controls were made by preparing the nutritive media in the same way as for corresponding biotic experiment, but without addition of neither alive nor killed cell biomass.

For *in situ* monitoring of Fe(III) citrate reduction, microbial suspensions were analyzed using X-ray absorption spectroscopy (XAS) at the French Absorption spectroscopy beamline in Material and Environmental sciences at ultra-high dilution (FAME-UHD) (Proux et al., 2005; Llorens et al., 2012) operating at the European Synchrotron Radiation Facility (Grenoble, France). Approximately 100 μL of the culture was loaded in a glassy carbon sample container developed to fit a high pressure/high temperature autoclave installed at the FAME-UHD beamline (Testemale et al., 2005). Based on epifluorescence microscopy counting, the cell density of the inoculum was ~3 · 10^8^ cells·mL^−1^ and ~6 · 10^8^ cells·mL^−1^ for *P. islandicum* and *T. scotoductus*, respectively. This setup and culture conditions were shown to be suitable to study iron reduction *in situ* in cultures of microorganisms under pressure and temperature characteristic of deep subsurface environments (Picard et al., 2015).

Iron K-edge (E_0_ = 7,112 eV) XAS measurements were conducted in fluorescence mode using a crystal analyzer spectrometer (Ge440 crystal with Bragg angle of 75.31°). The spectrometer was optimized to select the Fe Kα1 emission line of 6,405 eV. The monochromator energy was calibrated by setting the first maximum of the derivative of the spectrum of a Fe metallic foil at 7112 eV. This setup provided highly resolved XANES spectra and a detection limit in the range of a few ppm. The spectra were recorded in the range of 7,050–7,350 eV with 0.3 eV step at regular time intervals (~20 min). Several beam positions in the sample were measured to control the homogeneity in the recorded signal. The temperature set for the measurements was 98 °C and 65 °C (thermocouple temperatures) for *P*. *islandicum* and *T. scotoductus*, respectively, few degrees higher than the actual expected temperature inside the sample.

Spectra were processed using X-ray absorption visualization and analysis program Larix (version Larch 2025.2.0) (Newville and Ravel, 2024). The energy range relative to E_0_ was typically between −75 and −37.5 eV for baseline correction, and 25 and 220 eV for post-edge normalization. XANES spectra were fitted using a linear combination of 2 spectra of reference compounds: aqueous solutions of iron(II) chloride and iron(III) sulfate, both corresponding to hydrated Fe(II) and Fe(III) aqueous species. The fitting error of this linear combination procedure was less than 5%. Iron reduction rate was calculated using the following expression (Equation 2):


$$r_{Fe_{\mathrm{red}}}=\frac{k}{100}\cdot [Fe_{\mathrm{tot}}](\mathrm{mM}/h)$$ (2)

Where *k* is the slope of %Fe(II) vs. time (h), and *Fe_tot_* is the total Fe concentration (mM).

After the experiment, a 10 μL sample aliquot was placed onto a 0.22 μm polycarbonate filter for observation using scanning electron microscopy, as described above.

In separate experiments, Fe(III) citrate reduction was quantified using ferrozine assay (Phillips and Lovley, 1987). With care to avoid oxygen, samples were diluted in ultrapure water preliminarily acidified with H_2_SO_4_ (pH ≈ 2.5). Standards were prepared in the same solution using FeCl_2_ to obtain linear calibration in the range of 5–40 μmol/L (R^2^ = 0.999). 240 μL of 10 mmol/L ferrozine solution was added in 2 mL of diluted sample, pH was adjusted to ≈4.5 using ammonia-ammonium chloride buffer (pH = 10), and absorbance was measured within 10 min at 562 nm.

Several samples were characterized to verify ATP presence using luminescent ATP-assay (BioThema™) and C 110 lumitester (Kikkoman).

## Results

### Impact of microbial suspensions on the dissolution rates of basaltic glass

**Figure 2:**
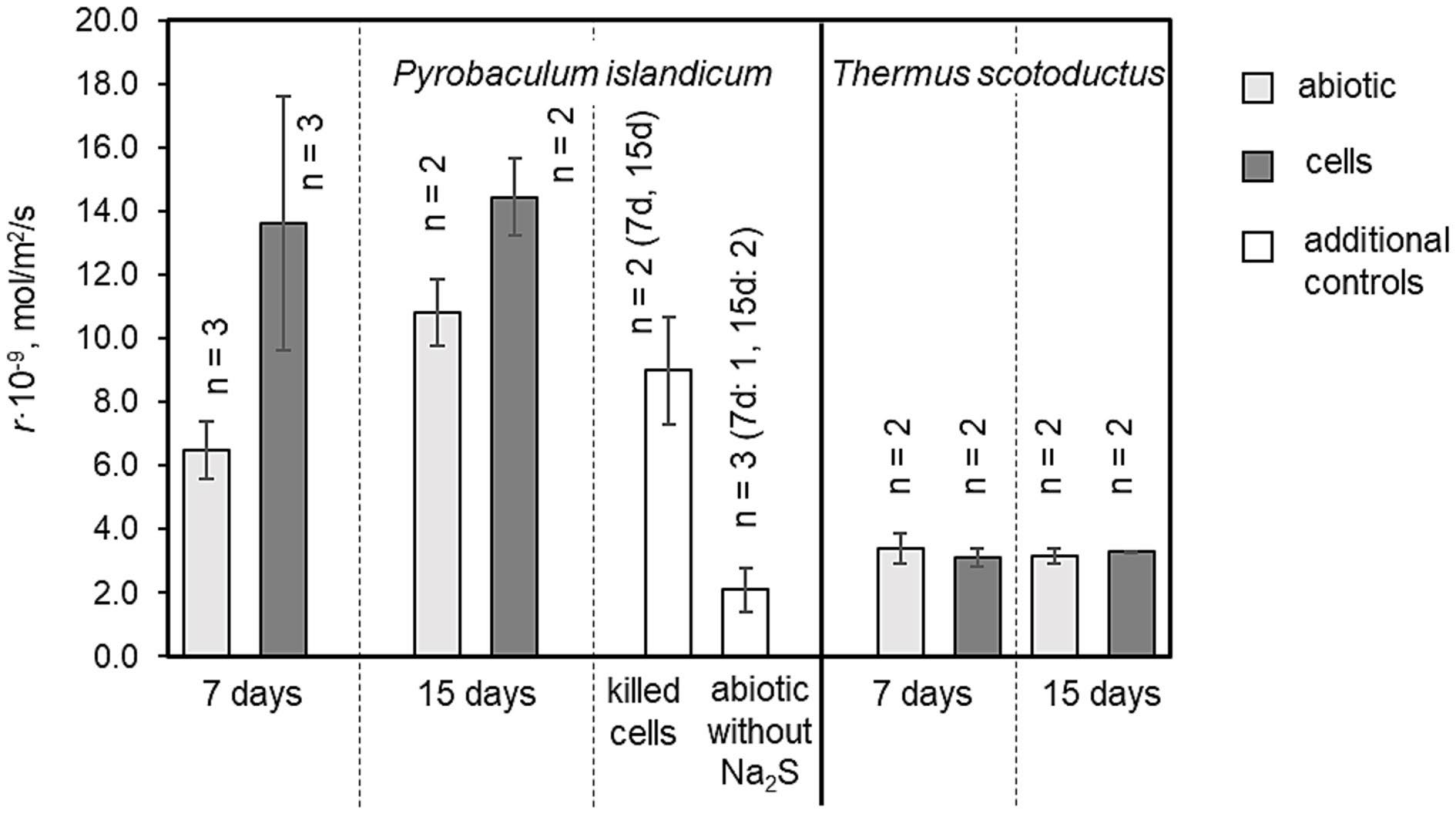
Dissolution rates of basaltic glass incubated in the nutritive media with (“cells”) or without (“abiotic”) microbial suspensions of *Pyrobaculum islandicum* and *Thermus scotoductus* for 7 or 15 days. Error bars represent standard deviations from *n* biological replicates, where *p* ≤ 0.01 for *Pyrobaculum islandicum* total abiotic (*n* = 5) and cell (*n* = 5) samples (Student’s *t*-test). Comparing 5 dissolution rates for living cells with 4 dissolution rates for killed cells, as listed in Supplementary Table S1, showed a significant difference between conditions (*p* = 0.016). Note that light-grey bars for abiotic samples of *Pyrobaculum islandicum* show samples that contained equal amounts of Na_2_S to biotic samples.

The incubation of the basaltic glass in microbial suspensions and corresponding abiotic conditions resulted in measurable surface retreats, from which dissolution rates were calculated (Figure 2). Overall, the glass dissolution rates measured in abiotic and microbial incubation media containing *T*. *scotoductus* at 60 °C were 2 to 4 times lower than those obtained with *P*. *islandicum* at 93 °C.

The exposure of basaltic glass to *T. scotoductus* microbial cultures did not lead to changes in the dissolution rates with respect to the abiotic controls neither after 7 days nor 15 days of incubation (Figure 2). *T. scotoductus* cells depleted of energy-reserve compounds to promote the use of basaltic glass for nutritive purpose, also did not enhance glass dissolution after 7-days incubation, as in the conditions without preliminary starvation (r_Thermus_starved_ = (2.7 ± 0.1) · 10^−9^ mol/m^2^/s vs. r_Thermus_normal_ = (3.1 ± 0.3) · 10^−9^ mol/m^2^/s).

During routine aerobic cultivation of *T. scotoductus*, we observed an accumulation of dark pigment in the cultures. To test whether this pigment displayed siderophore properties and promoted the dissolution of basaltic glass, basaltic glass was incubated with *T. scotoductus* cells at the beginning of aerobic culture growth. After 8 days, the calculated dissolution rate of basaltic glass was (1.8–2.0) · 10^−9^ mol/m^2^/s, compared to (2.7–2.8) · 10^−9^ mol/m^2^/s in the corresponding abiotic aerobic conditions. Thus, the presence of *T. scotoductus* pigment in the media did not lead to an increase of the dissolution rate of basaltic glass, indicating that the pigment of *T. scotoductus* is likely not a siderophore.

As opposed to *T. scotoductus*, *P. islandicum* cultures exhibited a modest yet consistent enhancement of basaltic glass dissolution, which was not seen in experiments with autoclaved *P. islandicum* cells (Figure 2; Supplementary Table S1). The difference in pH between the start and the end of the incubation was negligible (<0.5 pH units, Supplementary Table S1) and as such could not explain dissolution enhancement. Normalization to the cell content determined at the start of each biotic experiment also did not lead to a significant variation in the observed rates (Supplementary Figure S4). After 7 days, the surface retreat of basaltic glass exposed to *P. islandicum* was nearly twice that of the corresponding abiotic control. This difference, however, diminished over longer incubation times: after 15 days, the presence of *P. islandicum* led to only 1.3-fold increase in the dissolution rate vs. the abiotic control. Because Na_2_S, a strong reducing agent present in both biotic and abiotic *P. islandicum* samples, could contribute to basaltic glass dissolution, we conducted several experiments in degassed media without Na_2_S. We observed 80% decrease in cell count overnight in media without Na_2_S, which prevented continuation of the biotic experiment and indicated that the addition of a reducing agent was necessary to maintain cells viable during the incubation. In abiotic conditions without Na_2_S, the dissolution rate dropped markedly – from 6.5 · 10^−9^ mol/m^2^/s to 2.6 · 10^−9^ mol/m^2^/s after 7 days – and did not increase in longer experiments (Figure 2; Supplementary Table S1).

### Fe(III) citrate reduction rates in cultures with *Pyrobaculum islandicum* and *Thermus scotoductus*

**Figure 3:**
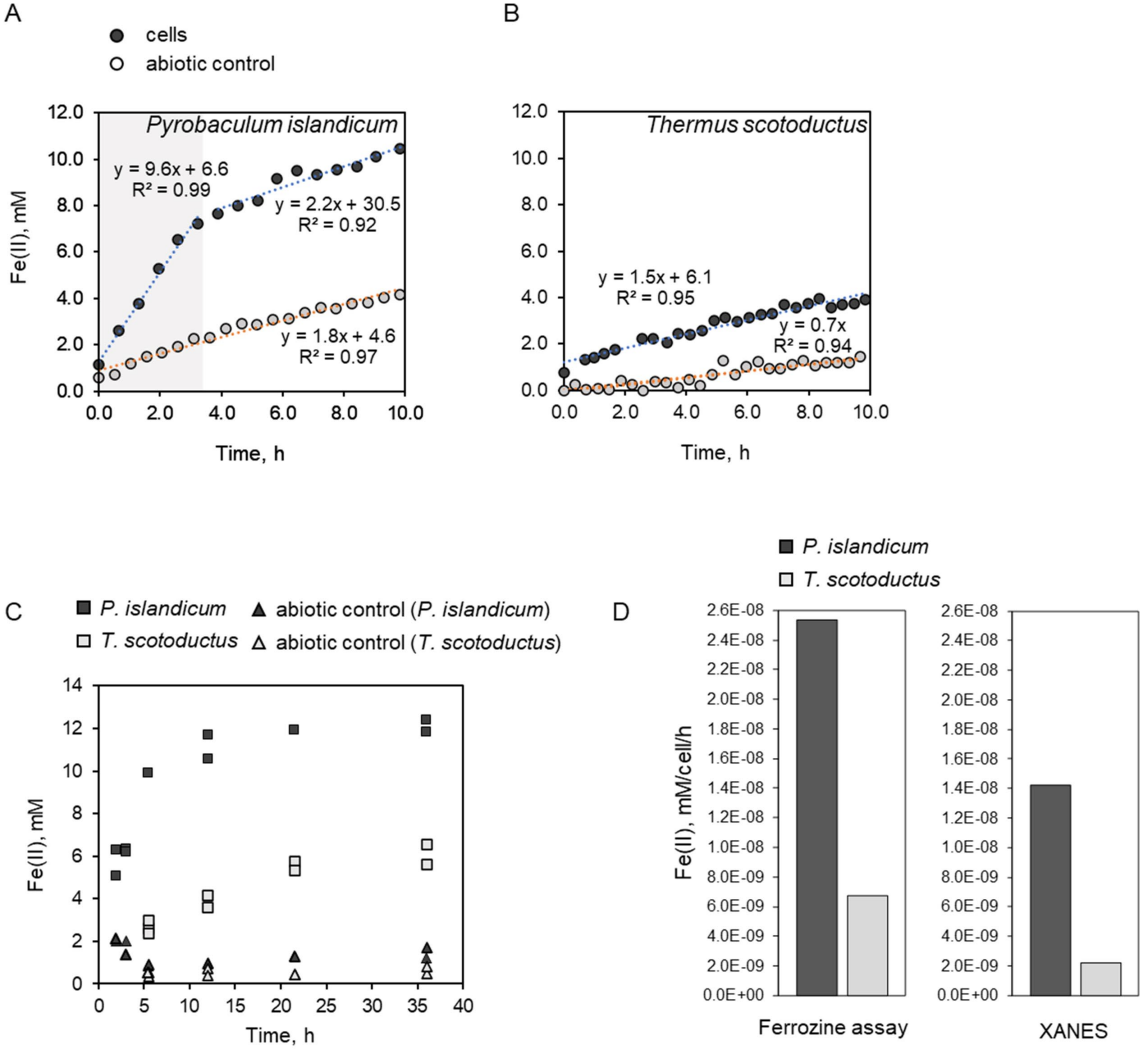
Fe(III) citrate reduction by *Pyrobaculum islandicum* and *Thermus scotoductus* determined based on **(A,B)** XANES *in situ* measurements or **(C)** *ex situ* incubations analyzed with ferrozine method. XANES linear fitting in (A) was performed by splitting the data into two regions, indicated by grey and white backgrounds on the graph. Data points in ferrozine assay in **(C)** correspond to separate biological replicates. **(D)** Fe(III) reduction rates per cell calculated in the first 6 h of incubation using ferrozine assay and XANES measurements.

To investigate if the potential of the microorganisms to enhance basaltic glass reactivity could be related to specific rates of microbial Fe(III) reduction, we quantified Fe(III) reduction rates using Fe(III) citrate as the iron source. The Fe(II) proportion from total Fe was determined using *in situ* XANES measurements and linear combination analysis of Fe(II) and Fe(III) reference compounds, as illustrated in Supplementary Figure S5. In *P. islandicum* culture, Fe(III) reduction was rapid during the first 3 h, with Fe(III) reduction rate approaching 2 mM · h^−1^ (Figure 3A). This trend was followed by the decrease in Fe(III) reduction rate down to 0.4 mM · h^−1^ from 4 to 10 h. After 10 h, the Fe(II) concentration reached ~10 mM. In contrast, in *T. scotoductus* culture, the kinetics of Fe(III) reduction was constant and relatively slow – approximately 0.3 mM · h^−1^ (Figure 3B). These values were higher than abiotic Fe(III) reduction rates – 0.4 mM · h^−1^ and 0.1 mM · h^−1^ for *P. islandicum* and *T. scotoductus*, respectively, indicating significant biotic contribution to Fe(III) reduction.

The results of the ferrozine assay (Figure 3C) show trends of Fe(II) accumulation in *P. islandicum* and *T. scotoductus* cultures similar to XANES data. We found ~11 mM and ~4 mM of Fe(II) in *P. islandicum* and *T. scotoductus*, respectively, after 12 h of incubation using ferrozine assay (Figure 3C). The rates of Fe(III) reduction observed during the first 5.5 h of the incubation were 2 mM · h^−1^ and 0.5 mM · h^−1^ in *P. islandicum* and *T. scotoductus* cultures, respectively. Thus, Fe(III) reduction was 4 to 6 times faster in *P. islandicum* cultures compared to *T. scotoductus*. When normalized to cell content, the comparison consistently supports the same trend (Supplementary Figures S6, S7 and Figure 3D). This suggests a very similar behavior of *P. islandicum* and *T. scotoductus* under conditions of the synchrotron experiment to *ex situ* incubations, demonstrating a high potential of XANES *in situ* measurements to monitor active biological process in real time.

The similarity of XANES and ferrozine assay results indicate that microorganisms were physiologically active under selected conditions of XANES spectra recording. Furthermore, the presence of ATP was confirmed in cultures exposed to X-ray beam using enzymatic assay and luminescence measurements. After the incubation at the synchrotron, *P. islandicum* and *T. scotoductus* cells were also observed using SEM on the filters with dried cell suspension aliquots (Figure S8). Many *P. islandicum* cells were homogeneously distributed on the filter, while *T. scotoductus* presence was less pronounced. This could be associated with longer total beam exposure time (14 h for *P. islandicum* versus 20 h for *T. scotoductus*), but also a clumping behavior of *T. scotoductus* cells also observed in the epifluorescence images of cultures before the experiment (Supplementary Figure S6b). Overall, the results of both XANES and ferrozine assays indicate high consistency between the two methods and show that *P. islandicum* exhibits a markedly higher Fe(III)-reducing capacity than *T. scotoductus* under the tested conditions.

### Chemical alterations and secondary phases on basaltic glass surfaces after incubation with *Pyrobaculum islandicum* and *Thermus scotoductus*

**Figure 4:**
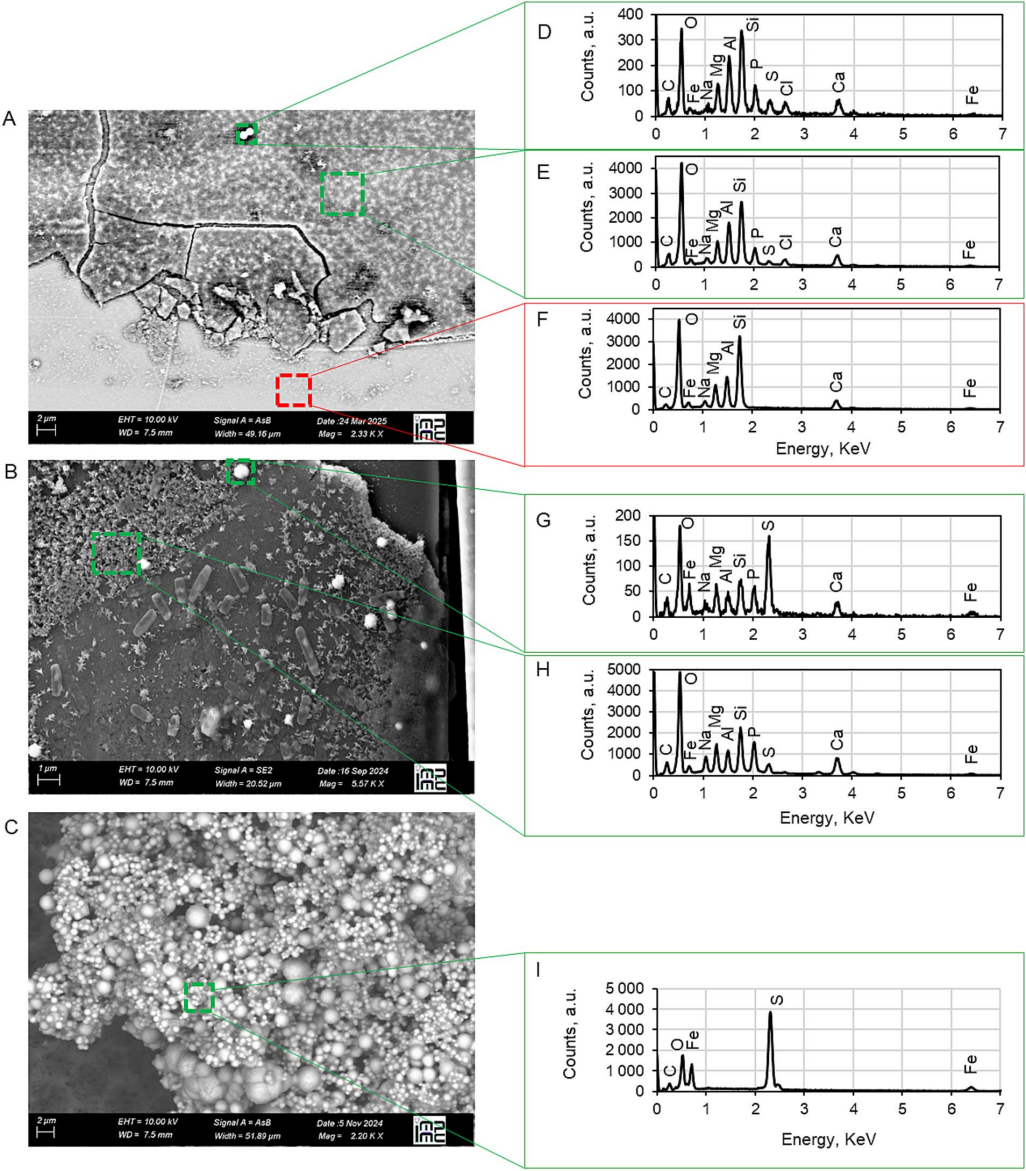
Chemical alterations in *Pyrobaculum islandicum* cultures: SEM images of basaltic glass **(A)** coupon and **(B)** grains exposed for 7 days to *Pyrobaculum islandicum*, and **(C)** secondary phases formed during incubation of *Pyrobaculum islandicum* in the medium with Fe(III) citrate without basaltic glass. SEM–EDX spectra in **(D,E,G,H)** correspond to the crust layer formed on the surface after the incubation and show increased relative intensity of Al, P, and S. SEM–EDX spectrum in **(F)** corresponds to the surface not covered by the crust layer. SEM–EDX spectrum in **(I)** corresponds to secondary structures of iron–sulfur-bearing minerals.

**Figure 5:**
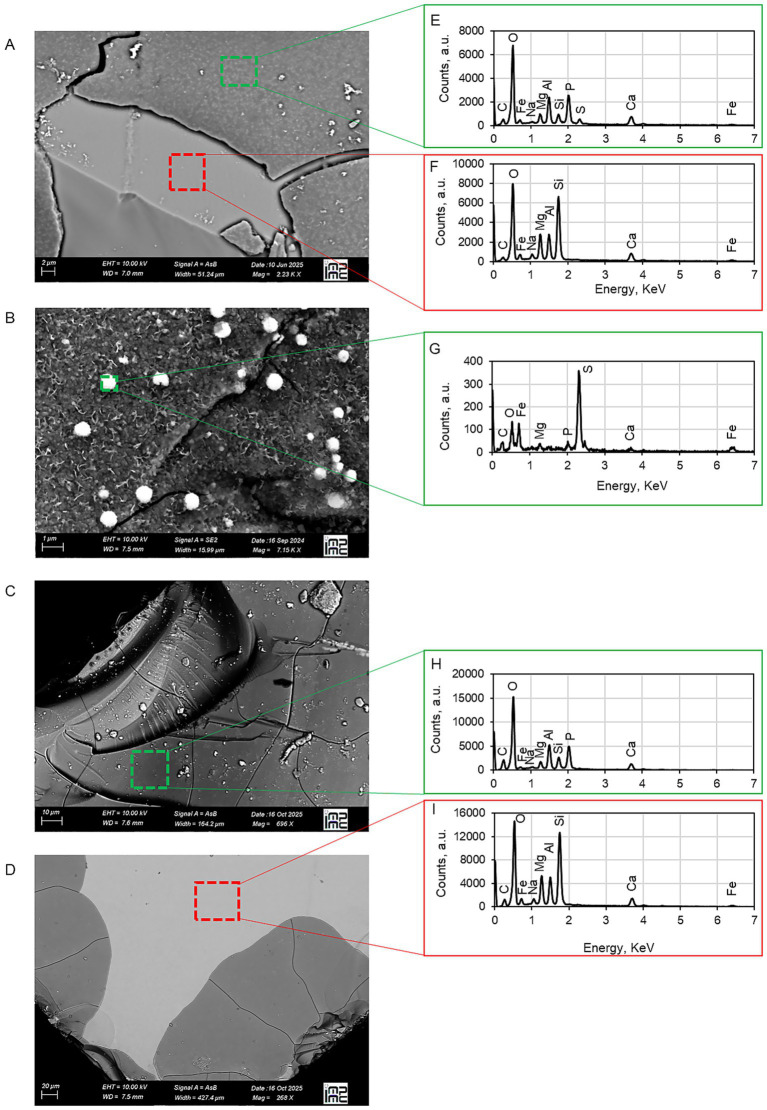
SEM images of basaltic glass **(A)** coupon and **(B)** powder, exposed for 15 days to **(A,B)** the nutritive medium with *Pyrobaculum islandicum*, and **(C,D)** the nutritive medium without *Pyrobaculum islandicum*. SEM–EDX analyses show the composition of basaltic glass incubated in **(E–G)** conditions with cells, or **(H,I)** conditions without cells. SEM–EDX spectra in **(E,H)** correspond to the crust layer and show increased relative intensity of Al, P, and Mg. SEM–EDX spectra in **(F,I)** correspond to the surface not covered with the crust layer. SEM–EDX spectrum in **(G)** corresponds to iron–sulfur-bearing minerals formed during exposure to *Pyrobaculum islandicum* cells.

Upon 7-days exposure of the basaltic glass to *P. islandicum*, several *P. islandicum* cells were present on the surface of the grains of the basaltic glass powder, but no biofilm was detected (Figure 4B). SEM observation of *P. islandicum* on the basaltic glass grains suggests the lack of extracellular matrix around the cells (Supplementary Figures S9C,D). In addition, secondary phases were observed on the glass surface (Figures 4A,B). These phases formed a crust layer that was partially detached during sample handling. The crust layer was enriched in Al, P, S, and occasionally Cl (Figures 4D,E,G,H), relative to parts of the surface where secondary phases were not present (Figure 4F). In addition, the layer included spherical particles rich in Fe and S (Figures 4D,G), similar to those formed after 7-days incubation of *P. islandicum* in the nutritive medium with Fe(III) citrate, where Na_2_S was added as a reducing agent. (Figures 4C,I). These particles were also observed on basaltic glass powders exposed to *P. islandicum* cultures for 15 days (Figures 5B,G). The EDX-analysis showed approximately two-fold higher S concentration relatively to Fe concentration in the particles (e.g., Figures 4G, 5G).

**Figure 6:**
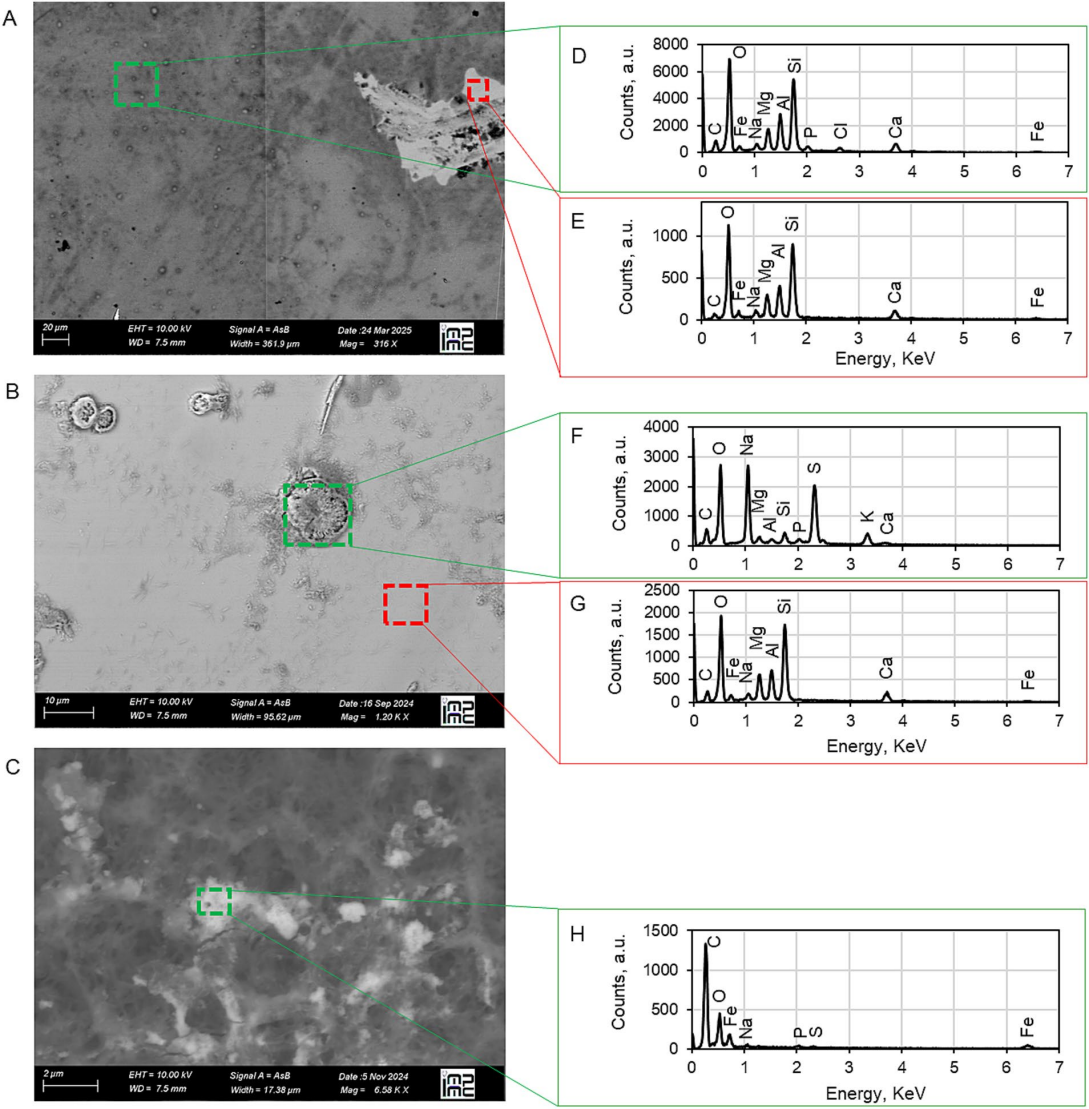
Chemical alterations in abiotic conditions corresponding to *Pyrobaculum islandicum* medium: SEM images of basaltic glass **(A)** coupon and **(B)** powder grain exposed for **(A)** 7 days and (B) 15 days to *Pyrobaculum islandicum* nutritive medium without cells, and **(C)** secondary phases formed during incubation of the medium with Fe(III) citrate without *Pyrobaculum islandicum* and without basaltic glass. SEM–EDX spectrum in **(D)** corresponds to the crust layer formed on the surface after the incubation and show increased relative intensity of Al, P, and Cl. SEM–EDX spectra in **(E,G)** correspond to the surface not covered by the crust layer. SEM–EDX spectrum in **(F)** corresponds to secondary structures assigned to Na_2_S. SEM–EDX spectrum in **(H)** corresponds to secondary particles observed after incubation of the medium with Fe(III) citrate without cells and without basaltic glass.

We also observed a crust layer on the surface of the basaltic glass exposed for 7 days to the nutritive medium without *P. islandicum* (Figure 6A). Relatively to the uncovered glass surface (Figure 6E), the layer was rich in Al, P, Cl, but lacked S in contrast to biotic conditions (Figure 6D). The relative content of Al and P in the layer formed under abiotic conditions was lower than in conditions with cells [atom (%) ratio Al/Si_bio_ = 0.42 vs. Al/Si_abio_ = 0.36, and P/Si_bio_ = 0.17 vs. P/Si_abio_ = 0.06, Supplementary Figure S10]. Higher P content in the glass layer of the biotic conditions compared to that of the abiotic ones was supported by SEM–EDX observation of cross-sections of the basaltic glass incubated for 7 days in abiotic or biotic conditions (Supplementary Figure S11). Supplementary Figure S11 also indicates the enrichment of Ca in the layer in both conditions relatively to the bulk glass, although Ca content was higher in the layer of the biotic sample.

The secondary phases observed on the grains of basaltic glass powder after incubation under abiotic conditions were rich in Na and S, suggesting possible Na_2_S precipitation formed after sample drying (Figures 6B,F). No strong evidence of iron–sulfur-bearing minerals formation was found in abiotic experiments conducted either with basaltic glass or Fe(III) citrate (Figures 6C,H).

**Figure 7:**
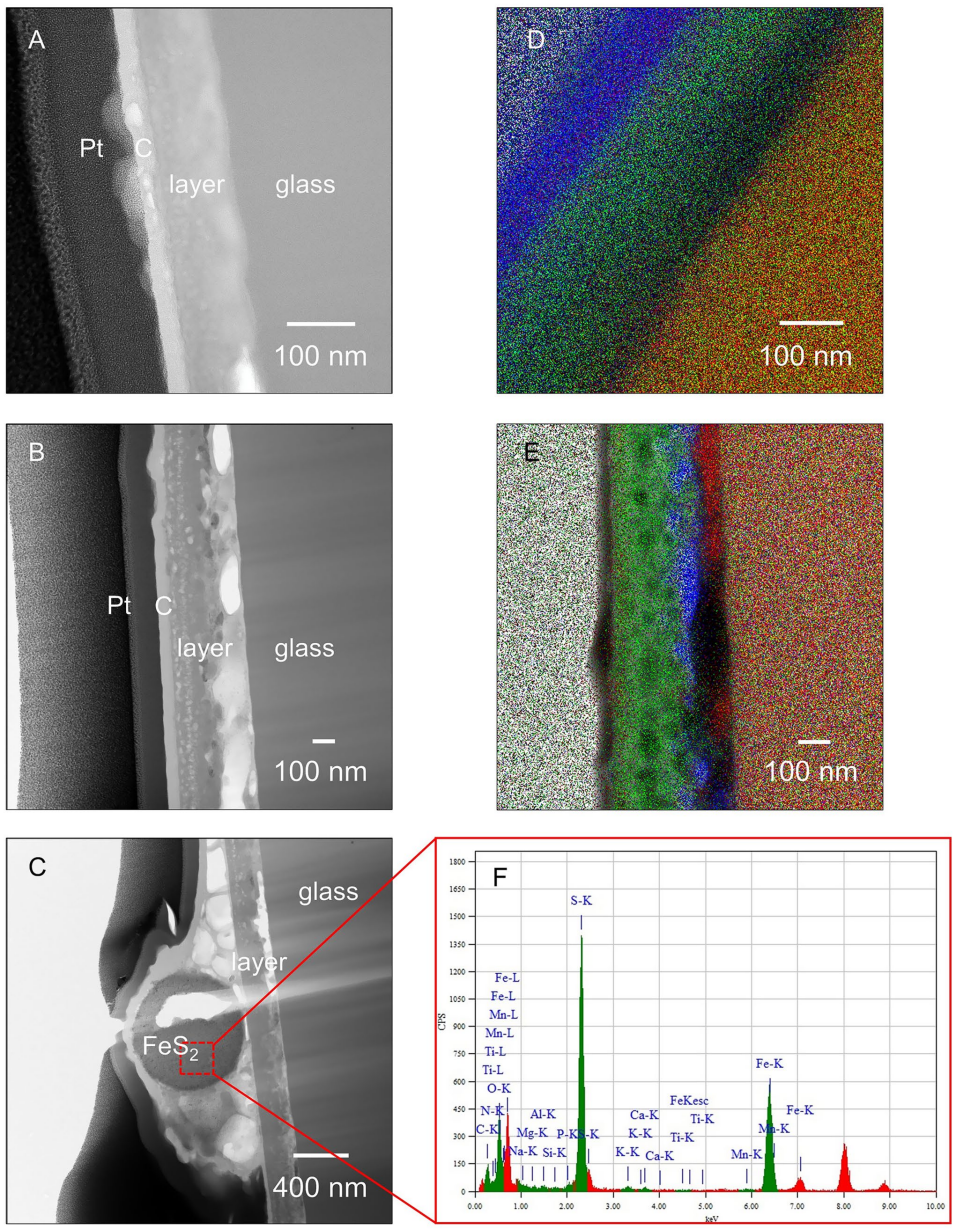
FIB-TEM analysis of basaltic glass coupon surfaces exposed for 7 days to **(A)** a nutritive medium without cells, or **(B,C)** to *Pyrobaculum islandicum*. Images in **(D,E)** correspond to STEM chemical composition maps for abiotic and biotic samples, respectively, where **(D)** carbon (blue), aluminum (green), and silicon (red), or **(E)** iron (blue), aluminum (green), and silicon (red) overlays are presented. The spectrum in **(F)** corresponds to a (Fe, S)-rich spherical particle.

FIB-TEM cross-sections revealed further differences in the composition and structure of the alteration layer of basaltic glass samples obtained in abiotic and biotic conditions (Figure 7). The thickness of the alteration layer was 110–120 nm (Figure 7A) and 300–350 nm (Figures 7B,C) in abiotic and biotic conditions, respectively. These results are in accordance with thin (~100 nm) uniform alteration layer and thick (up to ~400 nm) segregated alteration layer observed on abiotic and biotic samples’ cross-sections, respectively, from replicate experiments using SEM–EDX (Supplementary Figure S11). Furthermore, these values are close to surface retreats detected with VSI on basaltic glass after 7-days incubations and used for determining dissolution rates presented in Figure 2 (180 ± 25 nm and 311 ± 36 nm). As surface retreats were determined after cleaning of the surfaces from the alteration layers, or in breaches of the layer, the alteration layer thickness corresponded to a major portion of the surface retreat. Therefore, the dissolution of basaltic glass could be significantly influenced by the structure and composition of the altered surface.

STEM maps showed Al-rich layers in both abiotic and biotic samples (Figures 7D,E), in accordance with SEM–EDX results. Both layers also contained P, but its quantification was interfered by Pt signal in FIB-TEM. However, basaltic glass incubated in biotic conditions also was enriched with Si and Fe on the surface (Figure 7E). Formation of Fe-Si-rich layers on basaltic glass surface was unexpected under conditions of low Si concentration (Supplementary Table S1; Supplementary Figures S1,S2). These findings warrant further analysis (e.g., Fe redox state) in future studies. In addition to the alteration layer, Figure 7C shows cross-section image of (Fe, S)-bearing crystal presented in Figures 4A,D. FIB-TEM analysis (Figure 7F) confirmed the SEM–EDX data indicating the composition of the crystal resembling to that of pyrite (FeS_2_). Furthermore, electron diffraction pattern was similar to a previously observed pattern of the biogenically formed pyrite (Supplementary Figure S12) (Truong et al., 2023).

The results of incubation of the basaltic glass for 15 days in abiotic and biotic conditions were in accordance with the results of 7-days exposure. The crust layers formed under biotic and abiotic conditions were strongly enriched in Al and P (Figure 5A,C,E,H). The biotic crust layer contained S, whereas it was absent under abiotic conditions. The data of 15-days exposure also reveal Mg accumulation in the crust layer of the glass incubated with and without *P. islandicum*.

**Figure 8:**
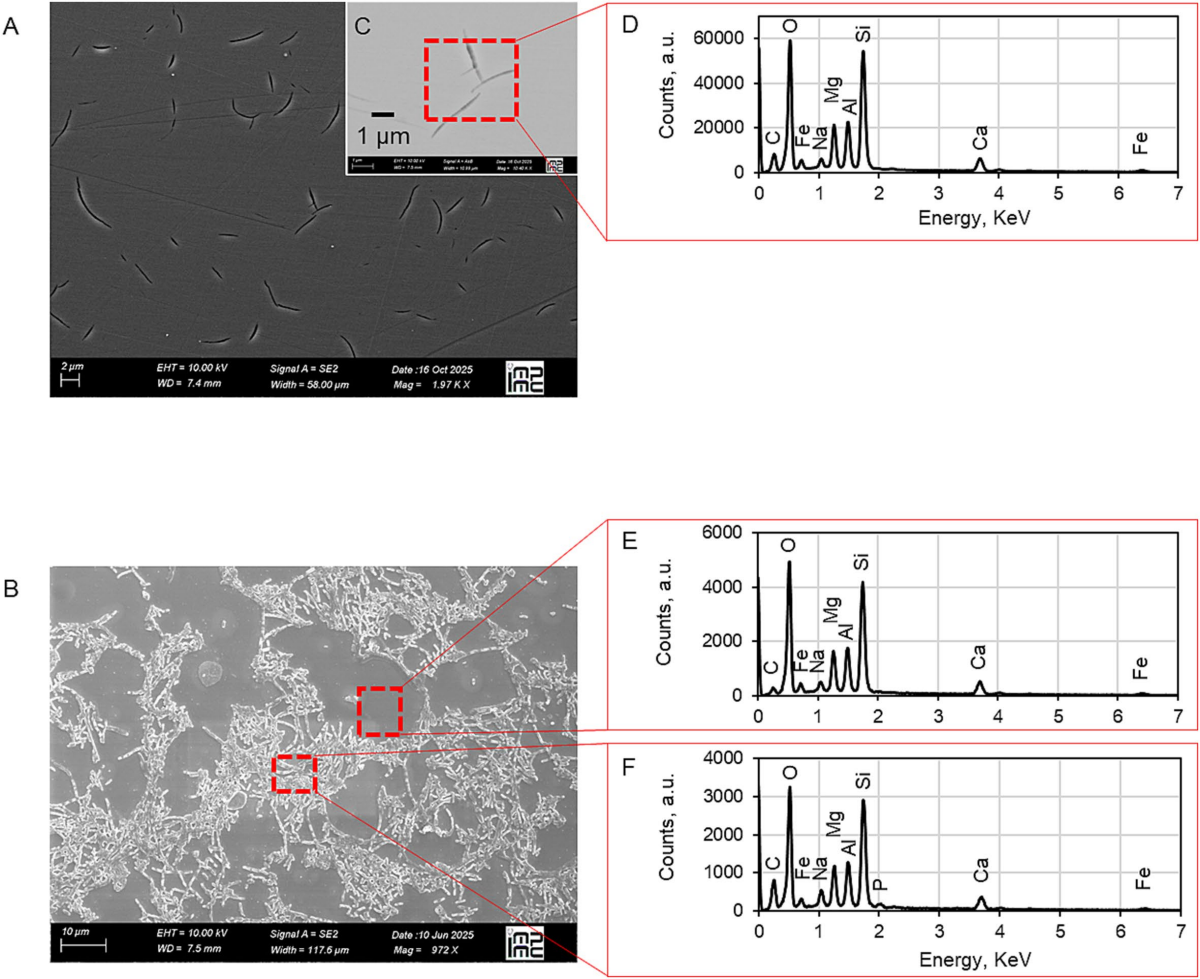
SEM images of basaltic glass exposed for 15 days to **(A,C)** the nutritive medium without *Thermus scotoductus*, and **(B)** the nutritive medium with *Thermus scotoductus*. SEM–EDX analysis shows the composition of basaltic glass incubated in **(D)** abiotic conditions, or **(E,F)** conditions with cells. SEM–EDX spectrum in **(E)** corresponds to the surface not covered by microbial cells. SEM–EDX spectrum in **(F)** corresponds to the surface covered by cells, and shows increased relative intensity of C, P, and Na peaks.

In contrast, no secondary phases were detected in experiments conducted in *T. scotoductus* medium either abiotic or inoculated with bacterial cells. Under both conditions, SEM–EDX analyses of basaltic glass surface after 15 days showed a composition similar to that of pristine basaltic glass (Figure 8, Supplementary Figure S11C,F). Biofilm formation was observed on the surface in experiments conducted with cells (Figure 8B). Etch pits were observed on the basaltic glass exposed to the medium without cells (Figure 8A and Supplementary Figure S13a). These pits were not compositionally distinct from the rest of the surface at the resolution of SEM–EDX (Supplementary Figure S14). The pits were not present in biotic conditions with cell concentration of 1.5–4.0 · 10^7^ cells·mL^−1^ (Supplementary Figures S13C,D). Reducing cell concentration down to ~0.5 · 10^7^ cells·mL^−1^ resulted in the appearance of dissolution pits (Supplementary Figure S13B). Therefore, it appears that the nutritive medium of *T. scotoductus* had a role in the formation of dissolution pits on the basaltic glass, which was inhibited by *T. scotoductus* cells. This result was supported by previously mentioned moderate decrease in the dissolution rate of basaltic glass in suspensions with preliminarily starved cells that formed denser biofilm, as shown in Supplementary Figure S15, compared to conditions without starvation. Overall, these findings confirm lower reactivity of basaltic glass in presence of *T. scotoductus* compared to that of *P. islandicum*. The formation of etch pits formed under abiotic conditions of *T. scotoductus* medium requires further investigation and is a scope of another study.

## Discussion

This study addresses the impact of Fe(III)-reducing microorganisms on basaltic glass reactivity. Below, we first compare the dissolution rates quantified under abiotic conditions in the nutritive media to results from the literature in diluted media to identify the impact of abiotic reagents. Medium composition (specifically, Si concentration), temperature, dissolved O_2_ concentration, and pH are major factors controlling basaltic glass dissolution and are therefore considered in the discussion (Guy and Schott, 1989; Berger et al., 1994; Oelkers and Gislason, 2001; Bas-Lorillot et al., 2026). Next, we isolate the contribution of *T. scotoductus* and *P. islandicum* to basaltic glass dissolution rate and propose underlying mechanisms. Finally, we offer perspectives for broader implications.

### Abiotic controls on basaltic glass dissolution

Abiotic dissolution rates were compared to those obtained by Bas-Lorillot et al. on the same glass in ultrapure water at 90 °C and 60 °C, where only LiOH or HCl was added for pH adjustment (Bas-Lorillot et al., 2026). After recalculating their rates to match the pH values of this study (~6.7 and ~7.5 for *P. islandicum* and *T. scotoductus*, respectively), dissolution rates in *P. islandicum* medium was 12 times lower than that in water, (6.5 · 10^−9^ mol/m^2^/s vs. 7.7 · 10^−8^ mol/m^2^/s) and 4 times lower in *T. scotoductus* medium (3.4 · 10^−9^ mol/m^2^/s vs. 1.4 · 10^−8^ mol/m^2^/s). Hence, the nutritive media used in the present study inhibited the dissolution of basaltic glass with respect to simple aqueous solutions.

In addition to pH and temperature, which were similar or identical in both studies, SiO_2(aq)_ and O_2(aq)_ were shown to affect basaltic glass dissolution rates (Bas-Lorillot et al., 2026). However, SiO_2(aq)_ and O_2(aq)_ concentrations were too low in the present study (Supplementary Table S1; Supplementary Figure S1, S2) to significantly inhibit basaltic glass dissolution.

Other factors potentially affecting the dissolution rate of basaltic glass include the solution salinity and the nature of cations, anions and ligands present in the media. Stillings and Brantley showed that, at acidic conditions, feldspar dissolution rate decreases in 0.01–0.1 M NaCl solutions compared to that in water because of the competition between H^+^ and Na^+^ for surface exchange sites (Stillings and Brantley, 1995). Conversely, Hausrath and Brantley showed that basaltic glass dissolution rate is not affected by CaCl_2_-NaCl-H_2_O solution at ionic strength as high as that of seawater (Hausrath and Brantley, 2010). Some studies even reported an increase in dissolution rate with increasing ionic strength for basaltic glass (Mesfin et al., 2023; Morin et al., 2015), quartz and amorphous silica (Dove and Nix, 1997; Icenhower and Dove, 2000). In this latter case, the mechanism proposed was the increase in frequency of Si–O bond hydrolysis in presence of solvated cations on silica surface. Hence, with the exception of the study by Stillings and Brantley, previous studies suggest that the increase in medium salinity does not lead to the inhibition of basaltic glass dissolution. We therefore conclude that the salinity of the incubation media in our experiments was not a factor contributing to an inhibiting effect of the nutritive medium on the dissolution of basaltic glass, and it is therefore unlikely that the difference in rates between the present study and that of Bas-Lorillot et al. is associated with the medium salinity (~3 g/L and ~5 g/L for *P. islandicum* and *T. scotoductus* medium, respectively).

Yeast extract and peptone present in the media could also play a role in basaltic glass dissolution, for instance by providing ligands for metal complexation. However, a study of basaltic glass dissolution in bacterial nutritive media containing yeast extract and peptone suggested that the presence of organic ligands limited Al and Fe precipitation and promoted stoichiometric release of all major elements from basaltic glass at pH 6–8 (Stockmann et al., 2012), without altering the dissolution rate of basaltic glass. Therefore, the presence of organic material in the nutritive media used in this study was likely not responsible for the relatively low dissolution rate observed.

Anions can influence the dissolution of basaltic glass by forming complexes with elements derived from basaltic glass network. Under basic conditions, Thorpe et al. reported that ~5 mM phosphate did not impact the dissolution rate of borosilicate glass (Thorpe et al., 2024). Under acidic conditions, the effect on glass dissolution rate seems to be anion-specific: while Al-sulphate complexes were suggested to increase basaltic glass dissolution rate (Flaathen et al., 2010; Dultz et al., 2016), the presence of 2.5 mM phosphate led to strong inhibition of initial nuclear glass dissolution rate (Gin et al., 2000), possibly because strong phosphate complexes with glass elements (e.g., Fe and Al) limited glass reactivity. Although phosphate adsorption is less favorable at circumneutral pH, it remains a plausible explanation for the relatively low dissolution rates observed here. Further dedicated experiments are required to corroborate this hypothesis.

Abiotic controls of *P. islandicum* discussed above, contained Na_2_S at the same concentration as the biotic incubation medium. Removal of Na_2_S from *P. islandicum* abiotic medium resulted in a nearly three-fold decrease in the dissolution rate of basaltic glass, and suppressed the rate increase observed between 7 and 15 days in the Na_2_S-amended abiotic controls. As a strong reducing agent, Na_2_S can enhance Fe(III) reduction, as demonstrated on various iron minerals (montmorillonite, lepidocrocite, ferrihydrite) (Rozenson and Heller-Kallai, 1976; Hellige et al., 2012; Zhang et al., 2023). As opposed to Fe(III), Fe(II) does not form polyhedra in a three-dimensional glass network and instead tends to cause depolymerization of these networks (Brown et al., 1995). Accordingly, basaltic glass shows slower dissolution rates in oxidizing conditions (Daux et al., 1997; Bas-Lorillot et al., 2026). Similar mechanisms were attributed to passivation of iron-silicate minerals (Schott and Berner, 1983; Wogelius and Walther, 1992; Saldi et al., 2013; Sissmann et al., 2013; Saldi et al., 2015). For instance, olivine dissolution rate was shown to increase when Fe(III)–Si-rich passivating surface layers are reductively transformed into Fe(II)–Fe(III) phyllosilicates (Saldi et al., 2015).

### Mechanisms of microbially-driven enhancement of basaltic glass dissolution

The key question underlying the present study was the extent of the biotic contribution to the dissolution of basaltic glass. We hypothesized that the microorganisms capable of Fe(III) reduction would enhance the dissolution following the mechanisms described for Fe-bearing silicate minerals at abiotic reducing conditions. We indeed observed an increase in basaltic glass dissolution rates in presence of *P. islandicum*: approximately two-fold after 7 days of incubation, relatively to abiotic controls containing Na_2_S. Hence, despite operating in a strongly reducing system containing Na_2_S, *P. islandicum* provided an additional enhancement to basaltic glass dissolution. *P. islandicum* can reduce several Fe(III) oxyhydroxide minerals, such as ferrihydrite, lepidocrocite and akaganeite, and to some extent, goethite, hematite, and maghemite (Kashyap et al., 2018). The extent of Fe(III) reduction by *P. islandicum* in these minerals correlates with the increase of mineral surface area and surface energy. Given that in basaltic glass, Fe(III) is integrated in a silicate matrix, its reduction is less favorable than that in iron oxyhydroxides, possibly explaining the relatively modest microbially-mediated enhancement of dissolution rate observed in the present study. Furthermore, studies on poorly crystalline Fe(III) oxyhydroxides suggested that *P. islandicum* requires direct contact with the surface in order to reduce Fe(III) on insoluble substrate (Feinberg et al., 2008). This fact could partly explain the decrease in biotic contribution to the dissolution of basaltic glass observed between 7 and 15 days in this study, given the formation of alteration layers, which impeded such a direct contact.

However, it should be noted that we did not detect localized dissolution pits that could be attributed to individual cells, or biofilm formation by *P. islandicum*. It therefore appears that the mechanisms of *P. islandicum* contribution to the glass dissolution could possibly include a change in the chemical composition of the medium resulting in the enhanced surface retreat. Closely related strains such as *P. aerophilum* and *P. arsenaticum* are capable of producing extracellular compounds to reduce Fe(III) from poorly crystalline Fe(III) oxyhydroxides (Feinberg et al., 2008). In parallel, it was reported that a change in the redox conditions of the medium can influence the interaction of *Pyrobaculum* species with minerals (Feinberg et al., 2008; House et al., 2002). For example, during incubation with basalt powder, *P. aerophilum* enhanced tungsten leaching under aerobic conditions but not anaerobically (House et al., 2002). In contrast, *P. islandicum* moderately increased tungsten leaching under anaerobic conditions, while the leaching of other elements (Fe, Ni, Cr) remained unaffected. Combining these previous results with the findings of the present study suggests the possible release of extracellular redox-active compounds by *P. islandicum* influencing basalt dissolution, and thus warrants further study.

Fe(III) reduction by *P. islandicum* promoted formation of iron–sulfur-bearing minerals on the surface of basaltic glass. The TEM analysis of these minerals revealed the composition and morphology previously observed in studies on *Thermococcales* and assigned to pyrite (Truong et al., 2023). The presence of pyrites on the basaltic glass exposed to *P. islandicum* cultures points to the reduction of Fe(III) derived from basaltic glass, since iron was excluded from all media and routine cultures. In addition, basaltic glass incubated in the medium with *P. islandicum* cells was covered with dark Fe-sulfide-like precipitates, uniformly covering the glass surface and the resin mask (Supplementary Figure S16). In parallel, we did not observe sulfur in the secondary precipitates formed under either abiotic conditions or in the presence of dead *P. islandicum* cells, and the surfaces stayed visually intact (Supplementary Figures S16, S17). Previous studies on *P. islandicum* with akageneite also evidenced sulfate minerals on the surface after the incubation with cells (Kashyap et al., 2023), suggesting that coupled Fe-S transformations may be important in the microbial alteration of basaltic glass, in relevance with environmental habitat of studied strains.

In accordance with these observations, the alteration layer of basaltic glass in *P. islandicum* incubations contained S, with S/Si increasing from 7 to 15 days of the incubation. Along with S, we observed strong enrichment in P and Al in secondary precipitates formed in presence of *P. islandicum* cells. Phosphates were previously observed when lepidocrocite and akageneite were incubated with *P. islandicum* (Kashyap et al., 2023). The formation of iron phosphate phases was also enhanced on the surfaces of synthetic remelted granite coupons incubated with *Paenibacillus* species capable of iron reduction (Parker et al., 2024). In our study, P and Al were also present in precipitates formed under abiotic conditions of *P. islandicum* medium, although to a lesser extent. Since Al is part of the glass network and not mobile at circumneutral pH, its presence in the secondary phases suggests initial dissolution of basaltic glass followed by a precipitation reaction. This observation is supported by several studies reporting formation of silica-rich alteration layers by a dissolution-precipitation process (King et al., 2010, 2011; Hellmann et al., 2012). The role of Al in enhancing dissolution has been reported for olivine (Sissmann et al., 2014; Andreani et al., 2013), possibly because of its incorporation in the silica-rich alteration layer. Cailleteau et al. suggested that the presence of insoluble elements in the alteration layer of glass limits the densification of silicate matrix and pore closure, thus increasing the glass reactivity (Cailleteau et al., 2008). In the present study, it is difficult to interpret at this stage how Al presence in the precipitation layer influenced glass reactivity. On the one hand, Al/Si ratio increased in the crust layer of the glass after 15 days of incubation compared to 7-days incubation in both biotic and abiotic conditions, along with the increase of glass dissolution rate. This finding indicates that Al in the surface layer did not inhibit dissolution, in agreement with previously mentioned studies. However, as described above, the dissolution rate of the glass found in this study was lower than that determined in deionized water. Since Al in the layer increases concomitantly with P, while P was shown to inhibit the glass dissolution in some cases (see above), the exact influence of the crust layer composition on the dissolution of glass in this study remains unresolved.

In contrast to effects observed in presence of *P. islandicum*, *T. scotoductus* did not alter the glass surface or increase dissolution. Yet *T. scotoductus* was reported to produce a pigment previously assigned to a melanin-like compound (Kristjánsson et al., 1994). Some melanins contain catechol groups that act as siderophores, and siderophores may enhance basaltic glass and silicate mineral dissolution (Perez et al., 2016; Buss et al., 2007; Valbi et al., 2023). Based on the lack of the visual change in color of the medium during the incubation of basaltic glass with *T. scotoductus*, the production of the pigment was limited. This may reflect nutrient limitation and low metabolic activity when basaltic glass served as the sole electron acceptor. Even under oxidizing conditions where highly metabolically active *T. scotoductus* cells synthesized the pigment, basaltic glass dissolution rate remained unaffected. The observed rates under biotic conditions represented only 70% of the abiotic rates under aerobic conditions. These findings indicate that the pigment of *T. scotoductus* is likely not a siderophore, and that *T. scotoductus* cells exert a net passivating effect, an effect analogous to microbially induced passivation reported for some bacterial strains on calcite (Stigliano Wild et al., 2025; Stigliano Benzerara et al., 2025). The interpretation is supported by (i) the decrease of the number of dissolution pits in presence of *T. scotoductus* cultures relatively to the nutritive medium without added cells, and (ii) starved cells forming high-density biofilm maintaining only 80% of dissolution rates observed with non-starved cells that formed less dense biofilm.

A possible limitation of the present study is that *T. scotoductus* was grown aerobically prior to the anaerobic incubation with basaltic glass. Microbial cells may require several consecutive culture transfers to adapt their metabolism to anaerobic growth and a faster growth on Fe(III). Nonetheless, *T. scotoductus* is commonly found in water containing low oxygen concentrations (Kristjánsson et al., 1994; Bas-Lorillot et al., 2025), and there is an increased evidence of O_2_ presence in groundwater and subsurface environments (Ruff et al., 2023, 2024). Therefore, fluctuations between aerobic and anaerobic metabolism could occur in the environmental habitat of this strain indicating the relevance of O_2_-containing pre-culture conditions.

The difference in the contribution of *P. islandicum* and *T. scotoductus* to the alteration of basaltic glass could be partly explained by the difference in the rates of Fe(III) reduction that we observed during incubation with Fe(III) citrate *in situ* and in real time using XANES measurements. These results were very similar to the results of the ferrozine assay obtained at several time points, demonstrating a high potential of XANES to monitor redox changes in live biological systems (Picard et al., 2012). A study of Salas et al. on ferrihydrite suggested that the alteration mineral products and their distribution are influenced by the microbial rate of Fe(III) reduction (Salas et al., 2010). Furthermore, as mentioned above, the abiotic *T. scotoductus* medium had less inhibiting impact on basaltic glass dissolution with respect to water, compared to that of abiotic *P. islandicum* medium. Accordingly, the impact of biotic reducing agent could be less pronounced in conditions where abiotic hydrolysis rates are already relatively high.

### Environmental implications

Following the discovery of the significant biotic contribution to Fe(III) reduction in sediments, soils and groundwater by mesophilic strains (Lovley, 1991), the last two decades have shown an increased interest in microbial Fe(III) reduction by thermophiles (Vargas et al., 1998; Amenabar and Boyd, 2019). Thermophilic and hyperthermophilic microorganisms contribute to mineral transformation in hydrothermal environments reflecting early Earth history and early forms of microbial life. The lack of oxygen in these systems promotes the growth of chemolithotrophic and chemoorganotrophic microorganisms capable of nutrient extraction from the mineral matrix (Rogers and Bennett, 2004; House et al., 2002). In silicate minerals, these microbially-mediated reactions can lead to the enhanced release of silica and other elements present in or adsorbed on the mineral (Vorhies and Gaines, 2009). The extent of microbial contribution to silicate weathering has important implications in modern-day strategies focused on carbon dioxide removal through carbonation reactions with silicate minerals. The two model microbial strains used in this study are relevant for such systems, as both strains belong to genera recently identified in borehole samples from the basaltic subsurface of Iceland, which is also considered for projects dedicated to carbon capture, utilization, and storage (CCUS) (Huber et al., 1987; Bas-Lorillot et al., 2025). The findings obtained on strains selected for our study indicate modest contribution of iron-reducing microorganisms to the dissolution of basaltic glass. However, the extent of biotic contribution by Fe(III) reducers may depend on the rates of enzymatic Fe(III) reaction and competing abiotic processes. Thus, in contexts where the concentration of abiotic reducing agents is low, the influence of Fe(III)-reducing microorganisms may be more pronounced.

The important finding of this study is the demonstration that *P. islandicum* promotes reduction of Fe(III) derived from the matrix of basaltic glass, a substrate far less favorable for electron transfer than Fe(III)-oxyhydroxides and highly relevant for environmental settings. *P. islandicum* is one of the most characterized species in terms of Fe(III) reduction in poorly crystalline and nanoscale iron oxide minerals, and this species is widespread in circumneutral to alkaline terrestrial hydrothermal environments (Feinberg et al., 2008; Amenabar and Boyd, 2019). The knowledge on the capacity of this strain to promote the reduction of Fe(III) in basaltic glass opens perspectives for further investigation of which biotic and abiotic factors control the rate of this process. Furthermore, we observed important changes in the glass alteration extent and products in presence of *P. islandicum* versus abiotic controls. These changes included (i) more rapid co-precipitation of aluminum and phosphorus in presence of cells, (ii) presence of sulfur in the secondary precipitates, and (iii) appreciable formation of iron–sulfur-bearing minerals assigned to pyrite. Collectively and on geological time scales, these processes can alter cycling of the involved elements, particularly iron and sulfur, abundant in volcanic subsurface environments (Amenabar and Boyd, 2019).

## Conclusion

The effect of *P. islandicum* and *T. scotoductus* on the dissolution rates of basaltic glass were investigated. *P. islandicum* contribution to dissolution was time-dependent: the rates increased two-fold after 7 days of incubation, but only 1.3-fold in 15-days incubations in presence of cells compared to abiotic conditions. The decrease in the enhancement of dissolution with time could be associated with formation of Al-, P-, and S-rich surface alteration layers thickening with time, preventing direct contact between microbial cells and the unaltered glass substrate. Iron–sulfur-bearing crystals assigned to pyrite were identified in the secondary precipitates formed in presence of *P. islandicum*. Conversely, basaltic glass showed no evident signs of alteration in *T. scotoductus* medium. *T. scotoductus* showed either no or modest inhibiting effect on basaltic glass dissolution. The differences between the impact of these two strains on basaltic glass dissolution could be related to differences in microbial Fe(III) reduction rates determined using incubations with Fe(III) citrate in this study. Our findings suggest that high rates of microbial Fe(III) reduction can likely contribute to factors promoting basaltic glass dissolution in subsurface environments.

## Data Availability

The original contributions presented in the study are included in the article/Supplementary material, further inquiries can be directed to the corresponding author/s.
