# Supplementary material for: Dissolution of Fe(III)-rich basaltic glass in microbial cultures of iron-reducing microorganisms
Source: Front Microbiol. 2026 Jun 1;17:1814551. doi: 10.3389/fmicb.2026.1814551 (PMC13265311; doi:10.3389/fmicb.2026.1814551)
Supplement: Supplementary file 1 [file Data_Sheet_1.pdf]

Supplementary information

Table S1. A summary of experimental conditions and results for incubations of basaltic glass

		Incubation time, h	Dissolution rate, mol/m^2/s	pH start	pH end	T, °C	O_2 conc., ppm	Si conc., ppm
Pyrobaculum medium (without cells) abiotic conditions	Sample 1	167	5.49E-09	6.6	6.6	93	-	0.8
	Sample 2	167	7.24E-09	6.7	6.7	93	-	1.2
	Sample 3	168	6.77E-09	6.7	6.7	93	-	1.1
	Sample 4	347	1.15E-08	6.7	6.8	93	-	2.4
	Sample 5	347	1.01E-08	6.7	7.1	93	-	3.6
Pyrobaculum medium (without cells) abiotic conditions without Na_2S	Sample 1	170	2.55E-09	6.1	6.1	93	0	1.0
	Sample 2	354	1.28E-09	6.1	6.0	93	0.2	1.2
	Sample 3	335	2.45E-09	6.1	5.4	93	0.6	1.5
Pyrobaculum medium (with cells) biotic conditions	Sample 1[a]	162	1.25E-08	6.8	7.7	93	-	4.2
	Sample 2	167	1.03E-08	6.8	7.1	93	-	0.9
	Sample 3	167	1.81E-08	6.8	n.m.	93	-	n.m.
	Sample 3	347	1.36E-08	6.7	7.0	93	-	2.9
	Sample 4	347	1.53E-08	6.8	6.8	93	-	2.8
Pyrobaculum medium (with killed cells) biotic conditions	Sample 1[c]	159	7.76E-09	6.8	7.1	93	-	1.6
	Sample 2[c]	159	7.89E-09	6.8	7.1	93	-	1.6
	Sample 3[c]	350	1.04E-08	6.9	7.3	93	-	4.0
	Sample 4[c]	350	9.95E-09	6.9	7.3	93	-	4.0
Thermus medium anaerobic condition (without cells) abiotic conditions	Sample 1	169	3.75E-09	7.5	7.5	60	0	2.0
	Sample 2	172	3.05E-09	7.4	7.4	60	0	1.7
	Sample 3	365	3.32E-09	7.6	7.5	60	0	2.9
	Sample 4	356	2.98E-09	7.4	7.4	60	0	2.8
Thermus medium aerobic condition (without cells) abiotic conditions	Sample 1[b]	185	2.68E-09	7.6	7.5	60	n.m.	5.4
Thermus medium anaerobic condition (with cells) biotic conditions	Sample 1	172	2.91E-09	7.5	7.5	60	0	2.0
	Sample 2[c]	165	3.44E-09	7.6	7.5	60	0	2.0
	Sample 3[c]	165	3.18E-09	7.6	7.5	60	0	2.0
	Sample 4	362	3.29E-09	7.5	7.5	60	0	2.4
	Sample 5[c]	362	3.29E-09	7.4	7.4	60	0	2.5
	Sample 6[c]	362	3.34E-09	7.4	7.4	60	0	2.5
Thermus medium aerobic condition (with cells) biotic conditions	Sample 1[b]	185	1.90E-09	7.6	8.1	60	n.m.	5.5
Thermus medium anaerobic condition (with starved cells) biotic conditions	Sample 1[c]	164	2.56E-09	7.6	7.6	60	0	1.7
	Sample 2[c]	164	2.76E-09	7.6	7.6	60	0	1.7

[a] Medium volume = 16.8 mL
[b] Medium volume = 20 mL
[c] Basaltic glass coupons replicates (two coupons of basaltic glass per incubation bottle) for one incubation time; all other samples represent individual bottles and freshly grown cultures
n.m. = not measured

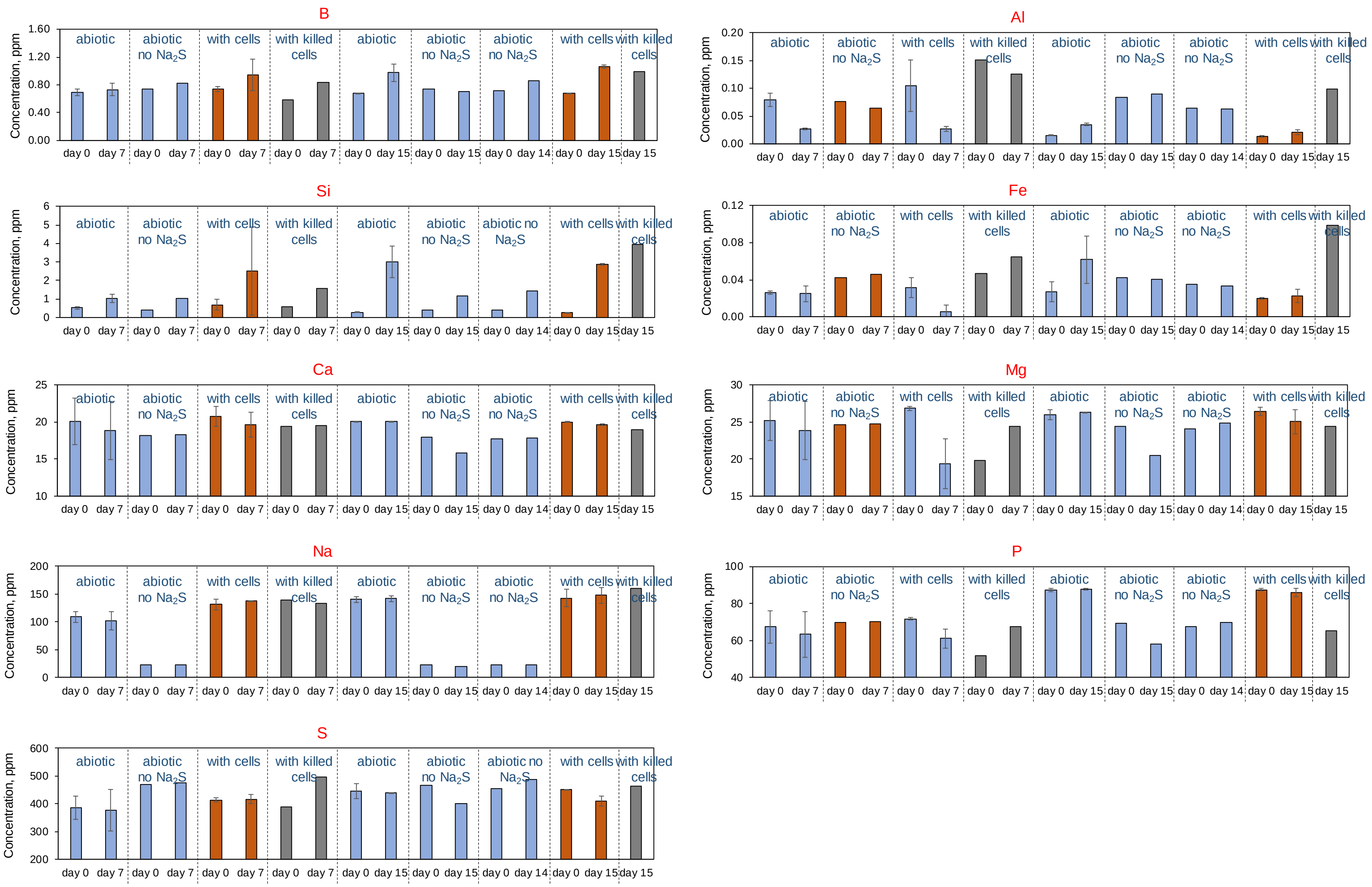

Figure S1. Major elements concentrations in *P. islandicum* solutions before and after incubations of basaltic glass in borosilicate glass bottles

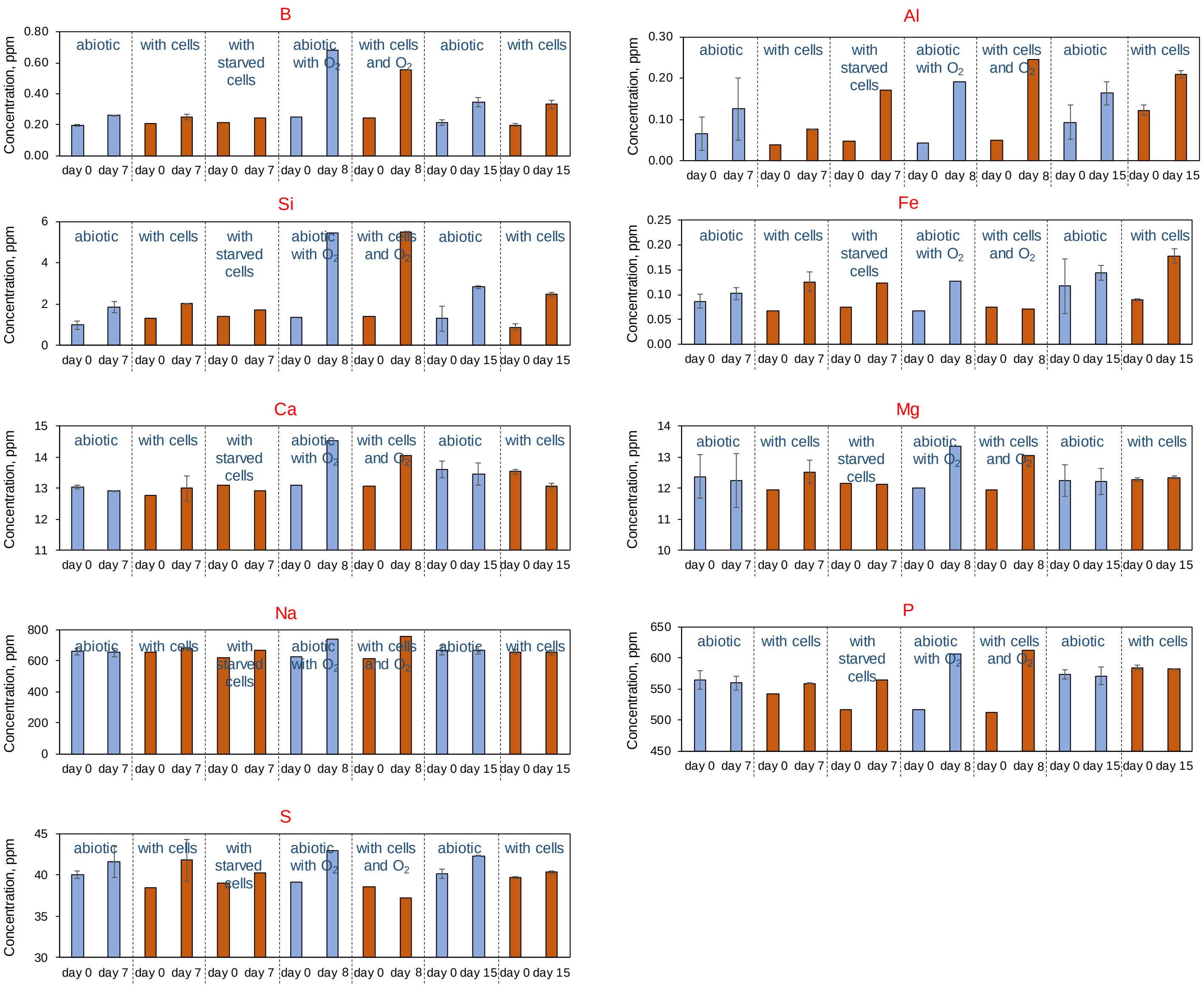

Figure S2. Major elements concentrations in *T. scotoductus* solutions before and after incubations of basaltic glass in borosilicate glass bottles

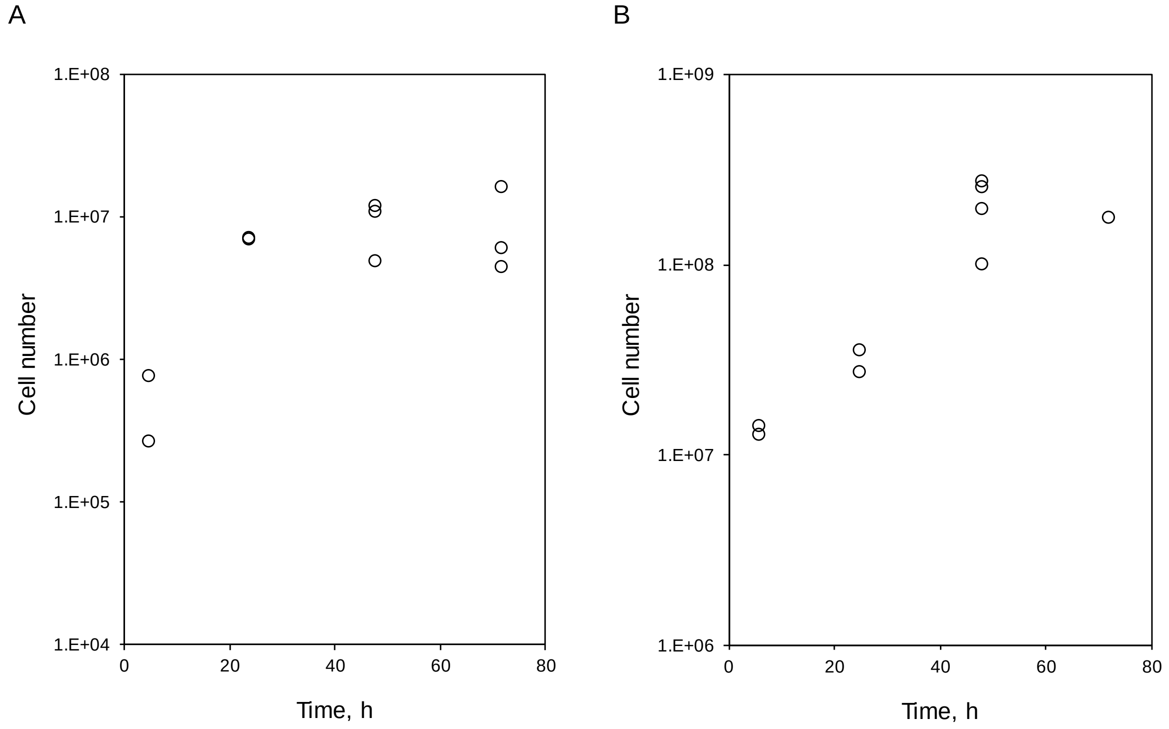

Figure S3. Growth curves of A) *P. islandicum* and B) *T. scotoductus*, where cell number was determined using flow cytometry measurements (see Materials and methods). Each point represents an individual sample.

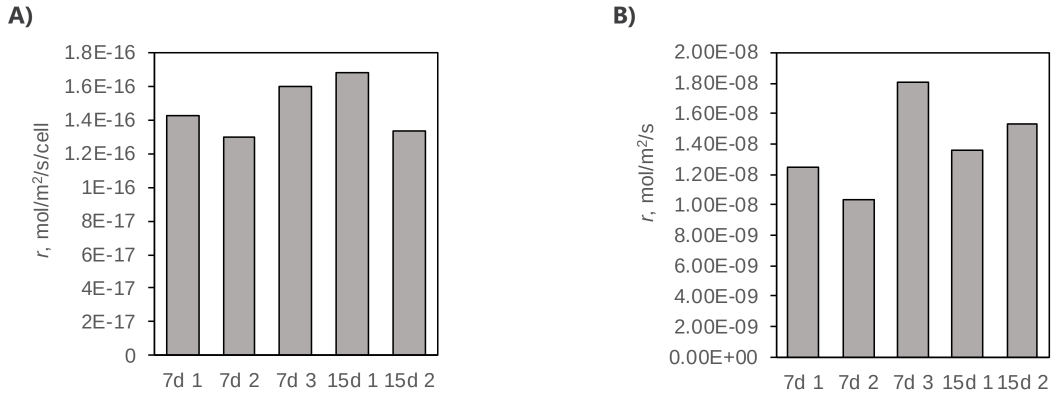

Figure S4. Dissolution rates of basaltic glass, A) with or B) without normalization to cell content at the start of the incubation, in the nutritive media with *P*. *islandicum* for 7 or 15 days. Each bar represents one biological replicate.

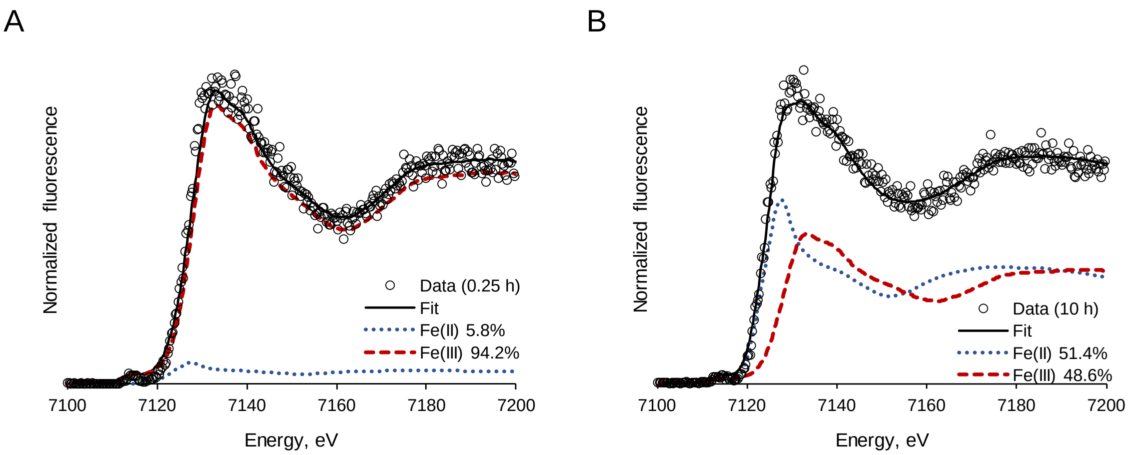

Figure S5. Linear combination fit of XANES spectra at the Fe K-edge XANES recorded at A) 0.25 h and B) 10 h during the incubation of *P. islandicum* at 98°C (temperature of the thermocouple).

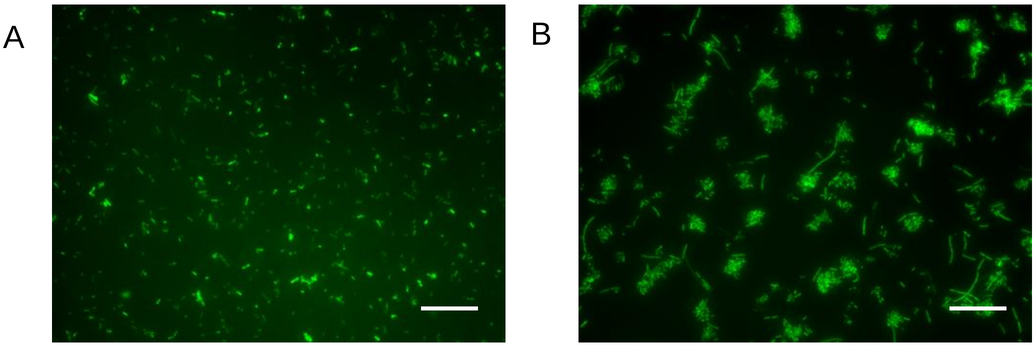

Figure S6. Representative epifluorescence images showing A) *P*. *islandicum* and B) *T*. *scotoductus* cultures used for Fe(III) reduction assay in XANES experiment, scale bar 20 µm

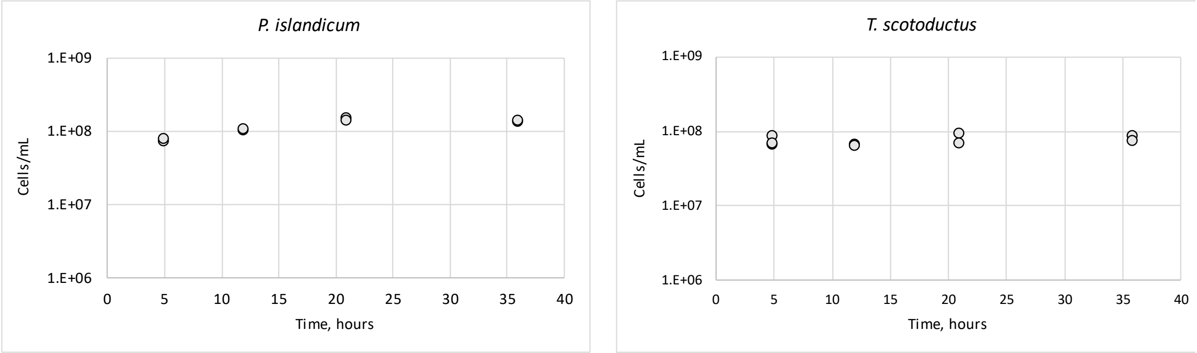

Figure S7. Cell numbers in cultures incubated with Fe(III) citrate and analyzed using ferrozine assay, in which each point corresponds to a separate biological replicate

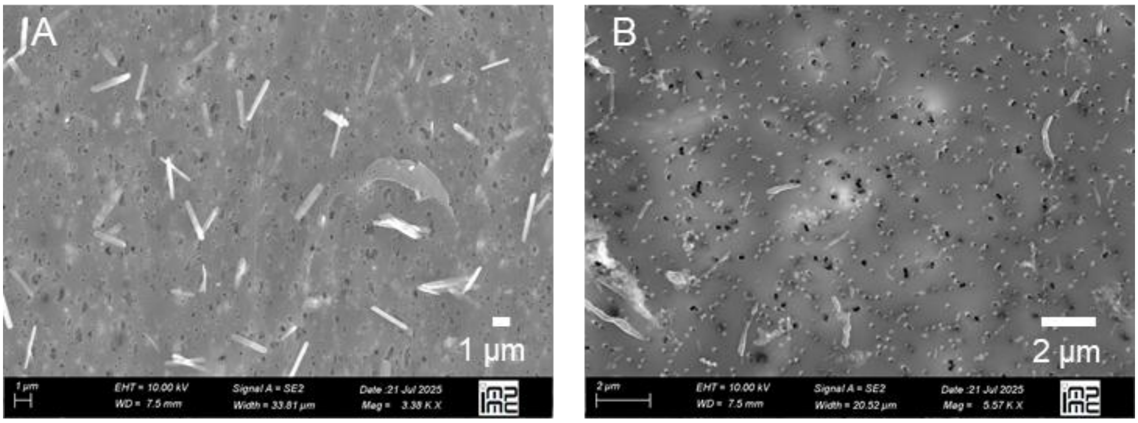

Figure S8. SEM images showing 10 µL aliquots of A) *P. islandicum* and B) *T. scotoductus* collected on the filters after XANES measurements performed for A) 14 hours or B) 20 hours

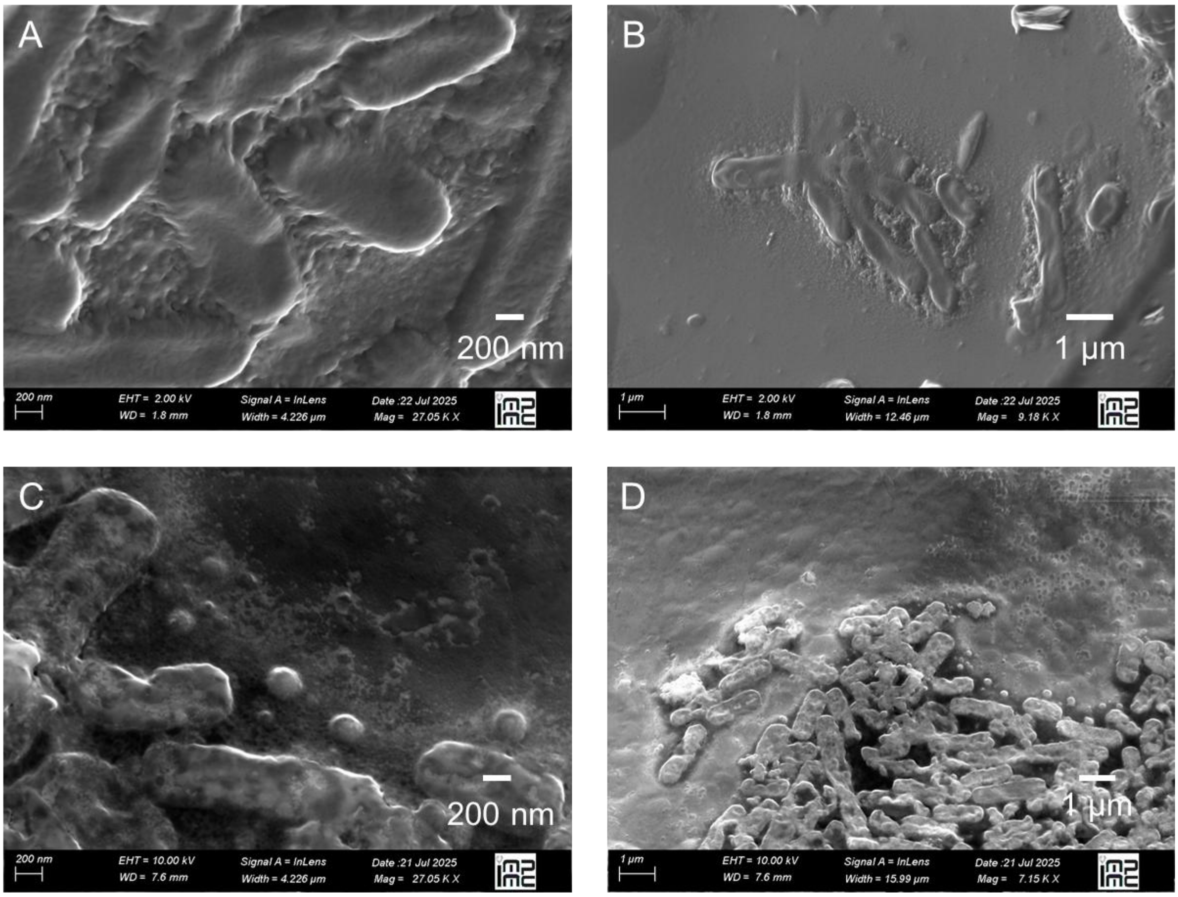

Figure S9. SEM images of A), B) *T*. *scotoductus* and C), D) *P*. *islandicum* on the basaltic glass surface after incubation for A, B) 23 hours or C), D) 18 hours

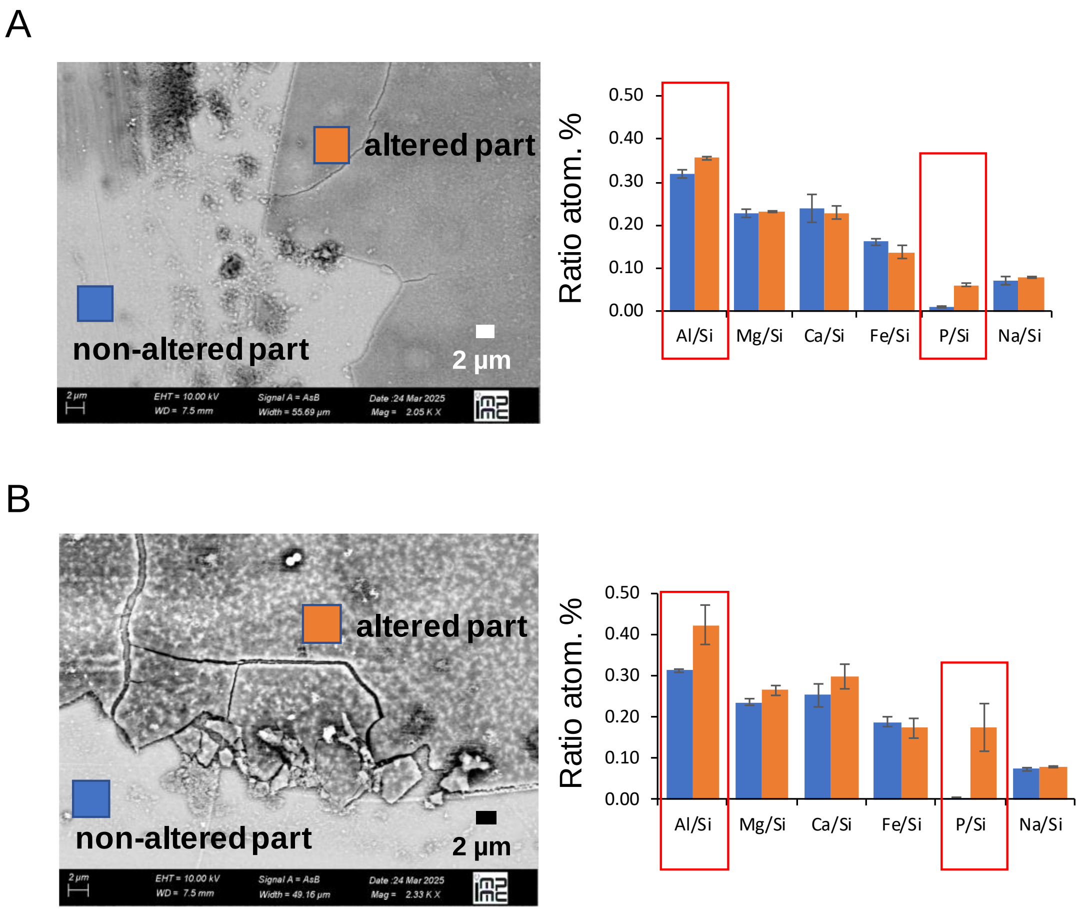

Figure S10. Atomic ratios of chemical elements corresponding to alteration layers or non-altered parts of basaltic glass surfaces, exposed to A) abiotic *P. islandicum* medium or B) medium with cells. Error bars represent standard deviation of ≥5 SEM-EDX maps for each condition. Red lines mark elements with most pronounced differences between abiotic and biotic conditions

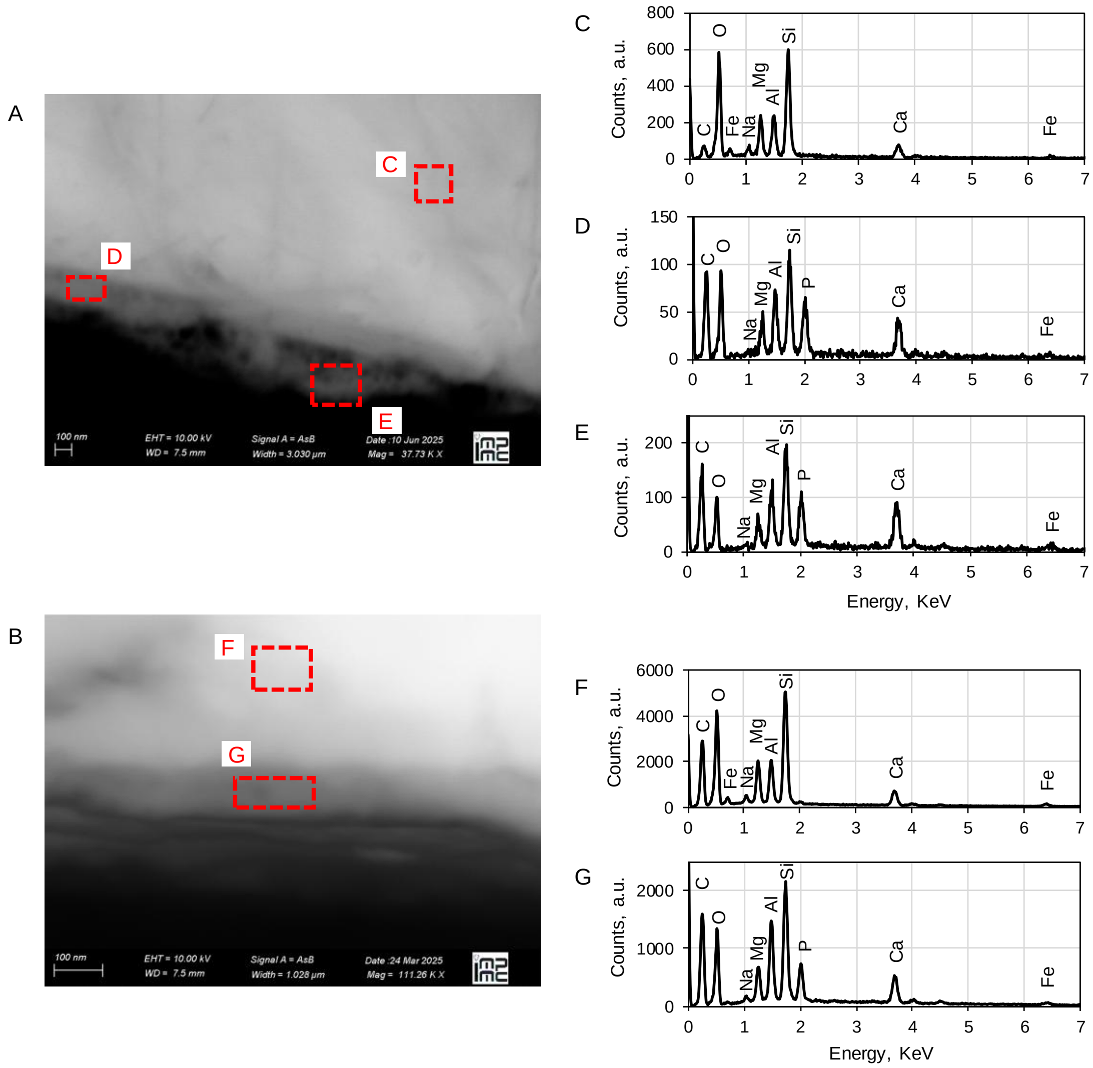

Figure S11. SEM images of the cross-sections of the basaltic glass exposed for 7 days to A) the nutritive medium with *P. islandicum*, and B) the nutritive medium without *P. islandicum*. SEM-EDX analysis shows the composition of basaltic glass incubated in C), D), E) conditions with cells, or F), G) conditions without cells. SEM-EDX spectra in C), F) correspond to the bulk glass. SEM-EDX spectra in D), E), G) correspond to the crust layer and show increased relative intensity of Al, P, and Mg

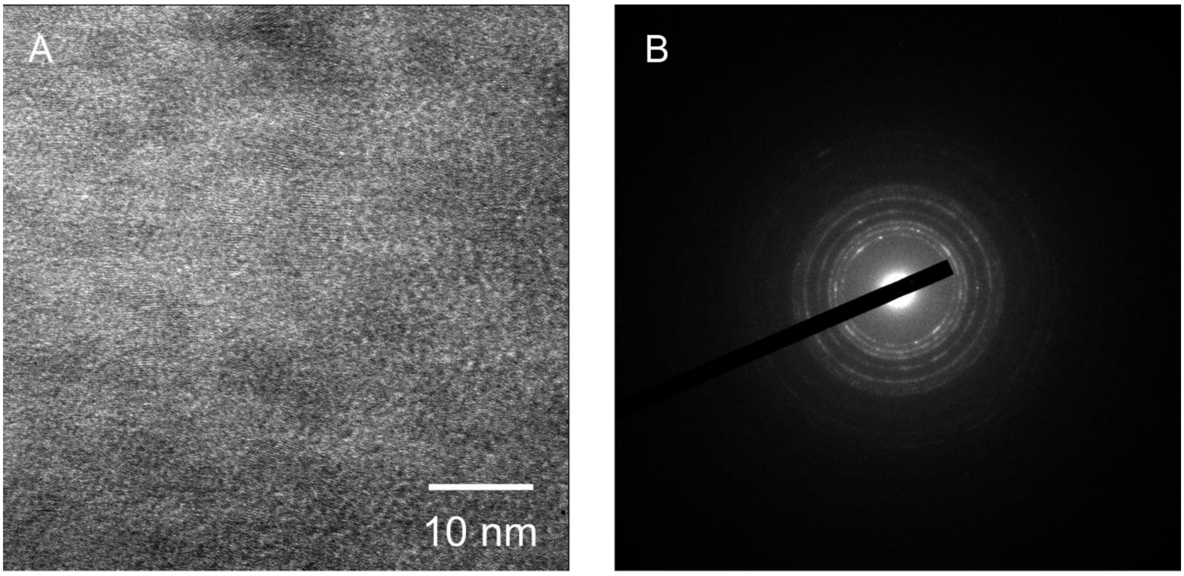

Figure S12. A) TEM image of (Fe, S)-bearing crystal showing atomic fringes, domain size ≈ 10 nm, as in pyrites synthesized by *Thermococcus kodakarensis* (Truong et al. 2023), and
B) corresponding electron diffraction pattern

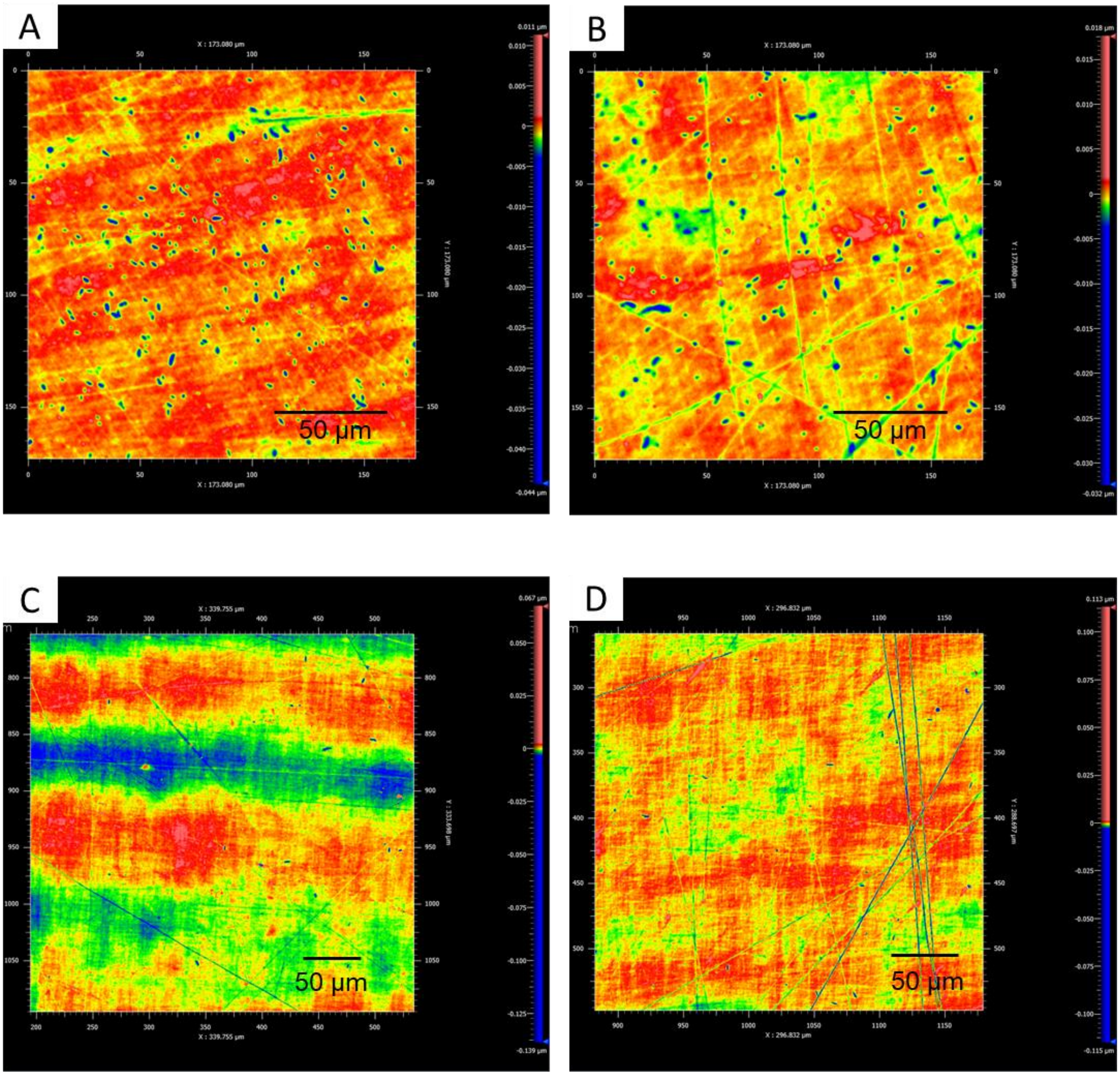

Figure S13. VSI images of basaltic glass surface after 7-day incubation in A) *T. scotoductus* medium without cells, or B) with cells at 0.5 · 10^7 cells·mL^{-1} (optical density at 600 nm = 0.02), C) with cells at 2–4 · 10^7 cells·mL^{-1} (optical density at 600 nm = 0.09), D) with preliminarily starved cells at 1.5 · 10^7 cells·mL^{-1} (optical density at 600 nm = 0.07)

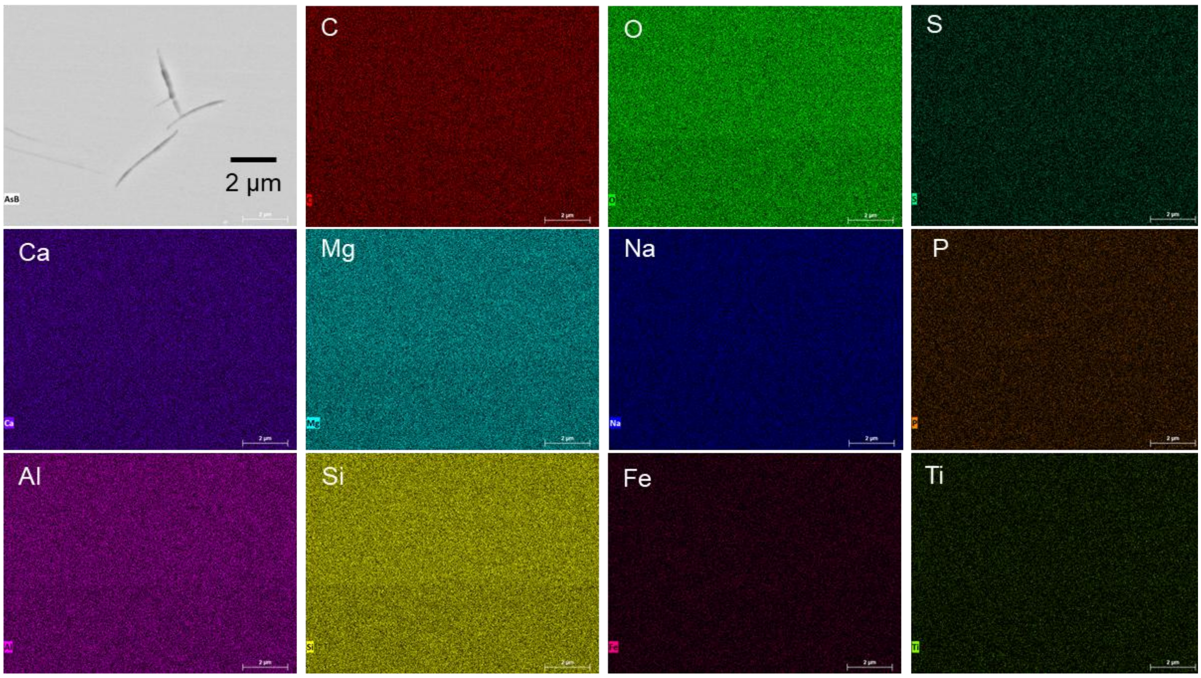

Figure S14. SEM-EDX analysis of basaltic glass surface after incubation in *T. scotoductus* medium without cells. Within the resolution of SEM-EDX, etch pits are not distinct in composition from the rest of the glass surface.

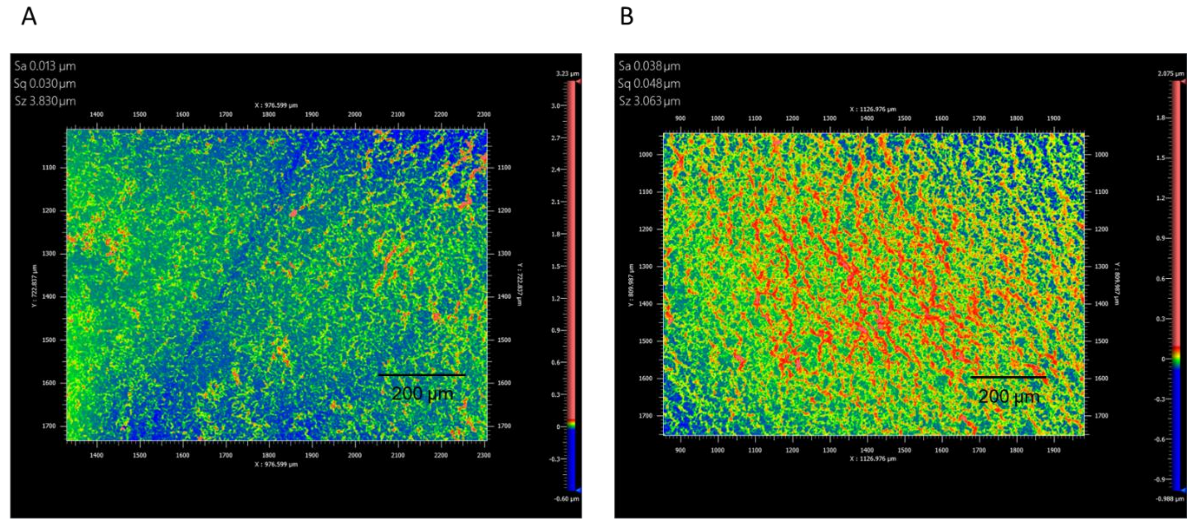

Figure S15. VSI images of *T. scotoductus* biofilms on basaltic glass surface incubated for 7 days in cell suspensions: A) cells were transferred to the medium with basaltic glass from the previously grown fresh culture, or B) starved in PIPES buffer before the incubation with basaltic glass

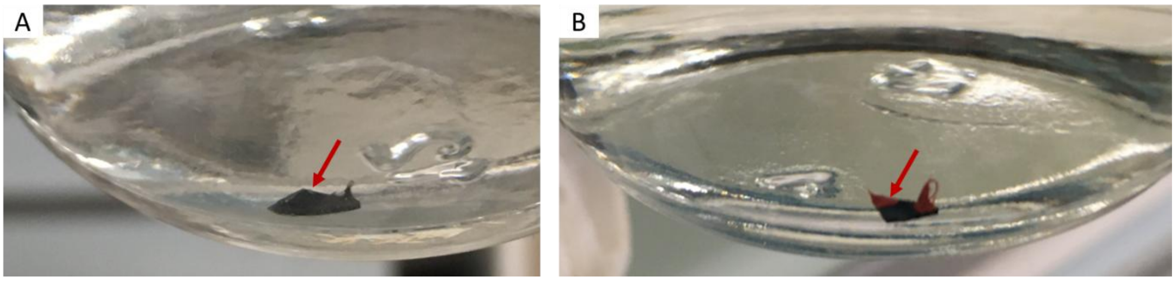

Figure S16. Photos demonstrating basaltic glass samples in the medium A) with *P*. *islandicum* and B) without, after 7 days of incubation at 93°C. Dark matrix covering the glass and the mask is demonstrated in A) and its absence in B).

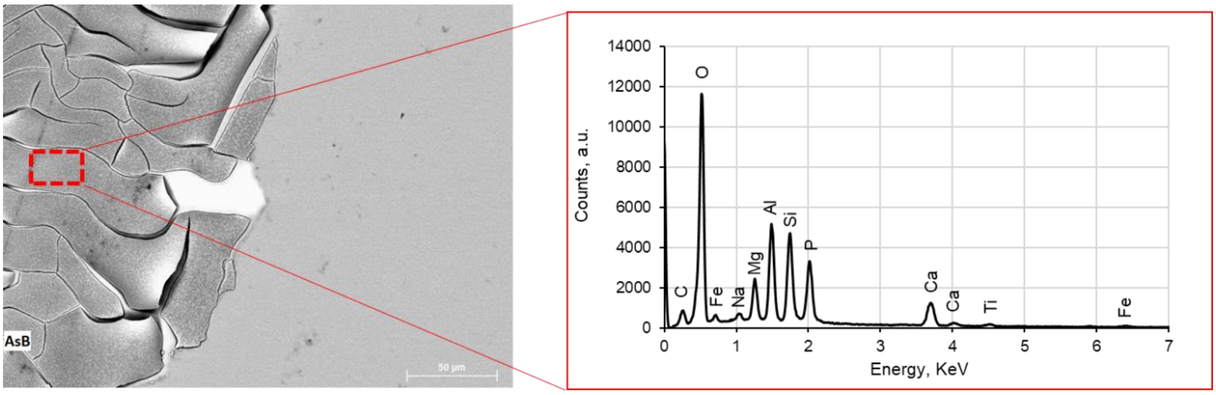

Figure S17. SEM image of basaltic glass exposed for 15 days to the nutritive medium with preliminarily autoclaved *P*. *islandicum*, and SEM-EDX analysis corresponding to selection in red
